# Fundamental Insights into Nanoconfined Devices by Using Electrochemical Impedance Spectroscopy

**DOI:** 10.1021/acs.analchem.5c07422

**Published:** 2026-01-23

**Authors:** Gregorio Laucirica, Danilo Echeverri, Gastón A. Crespo, María Cuartero

**Affiliations:** † UCAM-SENS, Universidad Católica San Antonio de Murcia, UCAM HiTech, Avda. Andres Hernandez Ros 1, 30107 Murcia, Spain; ‡ Department of Chemistry, School of Engineering Science in Chemistry, Biochemistry and Health, KTH Royal Institute of Technology, Teknikringen 30, SE-114 28 Stockholm, Sweden

## Abstract

Despite electrochemical impedance spectroscopy (EIS) being a powerful tool to inspect phenomena that occur at different time scales, its application to nanofluidic devices remains underexplored. In this study, we investigate the electrochemical behavior of glass nanopipettes internally coated with a carbon layer (CNPs, 60 nm radius) using EIS and electrochemical capacitance spectroscopy (ECS) to decouple ion transport from redox reactions. Through systematic measurements under varied conditions, we demonstrate herein that EIS and ECS can facilitate the estimation of key parameters, such as ion and electron transfer resistances, double-layer capacitance, and redox capacitance, by employing equivalent electrical circuits. Complementary methods, i.e., the distribution of relaxation times and differential capacitance, provide consistent values of resistances and capacitances compared to equivalent circuit analysis (<5% of difference between both methods) without predefined circuit assumptions, offering further insight into time constants and CNP resistance. Moreover, we underline the effectiveness of capacitance-based analysis in detecting redox species within the CNP domain at concentrations <10 μM, suggesting significant potential for sensing applications. Also, EIS effectively monitors surface modifications in the presence of nonredox-active compounds, as demonstrated using bovine serum albumin. Thus, protein adsorption led to a slight increase of 16 mV in peak separation in voltammetry; whereas EIS displayed a significant increase in both electron transfer resistance (from 0.88 to 18.4 MΩ) and ion resistance (from 8.55 to 10.6 MΩ). Overall, our findings underscore the potential of EIS in nanoscale electrochemistry, providing a valuable platform for fundamental studies and sensor development in nanoconfined domains.

## Graphical abstract

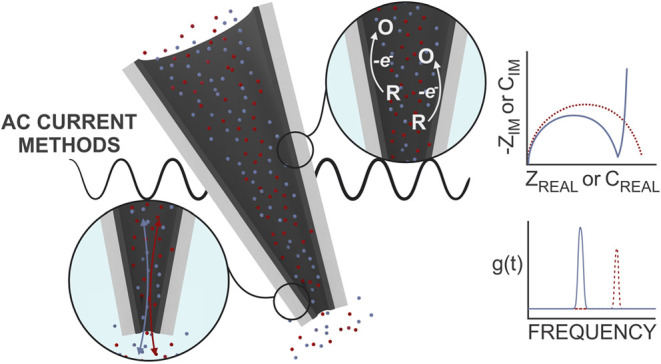

## Introduction

Electrochemical techniques have emerged as pivotal tools for investigating fundamental chemical processes and developing advanced materials for various applications, including sensors, energy storage, and clinical diagnostics. Among these techniques, electrochemical impedance spectroscopy (EIS) stands out due to its unique ability to provide insights into complex systems by characterizing both ionic and electronic transport phenomena.^1^ The fundamental principle of potentiostatic EIS involves perturbing the system with a small alternating voltage (input signal) over a defined range of frequencies, measuring the resulting sinusoidal current (output signal).^1–3^ The impedance (*Z*) of the system at different frequencies provides quantitative information on electron transfer, double-layer capacitance, and mass transport, allowing the separation of different processes based on their characteristic time constants.

The advancement of nanotechnology has led to the development of nanofluidic devices, where transport processes occur in confined geometries at the nanoscale.^4,5^ These devices, such as glass nanopipettes internally coated with a carbon layer (hereinafter referred to as carbon nanopipettes, CNPs), exhibit enhanced surface-to-volume ratios and unique interactions at their interfaces, which are critical in applications ranging from single-cell analysis to environmental monitoring.^6,7^ The electrochemical behavior of these nanoscale systems differs significantly from their macroscopic counterparts due to the dominance of surface effects and the increased significance of molecular interactions, driving interest in understanding and characterizing their dynamics.

To date, most studies on the electrochemical behavior of open CNPs have relied on direct current techniques, such as cyclic voltammetry (CV) and chronoamperometry.^8–11^ These techniques have revealed two critical aspects: (i) when open CNPs are immersed in any solution containing a redox agent, the liquid is allowed to enter the CNP tip by capillarity. The relatively large surface area of the nanopipette compared to the confined volume (in the pL range) creates a thin-layer regime, where redox analytes can be fully depleted within the typical time scales of electroanalytical techniques (in the order of seconds); and (ii) ion transport through the CNP aperture strongly influences the electrochemical response, indicating an association between ion and electron transfer processes. In this context, EIS is useful for exploring the interplay between ion transport and redox reactions due to their different characteristic frequencies.^11^ Effectively, it is possible to gain quantitative insights into ion transport kinetics and electronic contributions in redox processes at the nanoscale. This can be particularly valuable for refining CNP performance and tailoring their properties for specific applications. However, unlike conventional electrochemical techniques, extracting quantitative information from EIS is challenging because of the high level of complexity in interpreting the associated data.^3^

A typical method for analyzing EIS data is fitting the experimental impedance response to an equivalent electrical circuit model (ECM) to obtain various interesting parameters, such as resistance and capacitance.^12^ Nonetheless, this approach requires a profound understanding of the system, and in some cases, multiple ECMs may fit the same impedance spectrum, making their interpretation difficult.^13^ To overcome these issues, alternative data analysis methods have emerged, including the distribution of relaxation times (DRT).^3,14^ In this approach, the impedance spectra are modeled as time scale distribution functions (*g*(τ)), where the resulting plot displays discrete peaks that reveal the characteristic time constants of the involved processes. In contrast to ECM fitting, the DRT method does not require prior assumptions about the circuit diagrams, allowing for the deconvolution of overlapping electrochemical processes and the determination of the associated resistance value. Notably, DRT has gained interest due to its ability to (i) resolve processes with similar characteristic times, (ii) provide additional insights that can inform or refine ECM modeling, and (iii) extract resistance values without relying on predefined ECMs.^15,16^ Despite these advantages, the application of the DRT method beyond traditional energy storage systems (e.g., batteries and fuel cells) has been relatively unexplored.^17^

One limitation of the DRT method is its requirement for a convergent imaginary impedance at low frequencies (i.e., *Z* must be bounded).^3^ This condition is not met in systems exhibiting diffusion-limited transport or dominant low-frequency capacitances, restricting the application to high and moderate frequency ranges. To address this, alternative time-distribution-based methods have been developed.^18–20^ A notable example is the distribution of differential capacitance (DDC), which is particularly suitable for analyzing the low-frequency tail in capacitive behaviors, a trend that cannot be meaningfully captured by the DRT. Similar to DRT, DDC enables the extraction of quantitative information on characteristic times and capacitance values without requiring prior assumptions.^21^

Beyond fundamental aspects, EIS has also garnered attention in electrochemical sensing applications, where recognition events at the electrode surface induce measurable changes in electron transfer resistance (*R*_CT_) or capacitance.^22,23^ This capability allows for detecting biomolecular interactions, even in the absence of redox-active species. A particularly promising approach is the called electrochemical capacitance spectroscopy (ECS), which converts impedance data into a capacitive spectrum.^24,25^ ECS has demonstrated significant advantages, especially in terms of sensitivity, for developing affinity biosensors such as immunosensors, genosensors, and aptasensors.^26–28^ These biosensors operate by monitoring changes in the interfacial capacitance at the electrode–solution interface that occur upon specific biorecognition events. The binding of the target analyte alters the local dielectric properties and charge distribution, leading to measurable variations in the capacitive response.

Despite EIS demonstrating to be a powerful tool in traditional electrochemical systems, its application to nanofluidic device-based electrodes, such as CNPs, remains largely unexplored. Herein, we aim to bridge this gap by investigating the electrochemical behavior of open CNPs with EIS and ECS (Figure 1). By comparing EIS with CV experiments, we underline the advantages of EIS in disentangling ion transport from electron transfer phenomena. Additionally, transforming impedance data into a capacitive framework via ECS, we provide quantitative and absolute insights into the concentration of redox-active species, contributing to a deeper understanding of the nanofluidic electrochemical behavior. To reach even more comprehensive information, we employed ECM, DRT, and DDC analyses, which offer both qualitative and quantitative insights into the mechanisms governing the electrochemical response. Overall, these findings not only pave the way for advanced detection strategies in confined environments but also underscore valuable insights that can be derived from EIS when combined with various analytical methodologies. As an example, our results demonstrate that EIS in conjunction with ECS, DRT, and DDC can effectively amplify subtle surface modifications, even those involving nonredox active species such as proteins.

**Figure 1:**
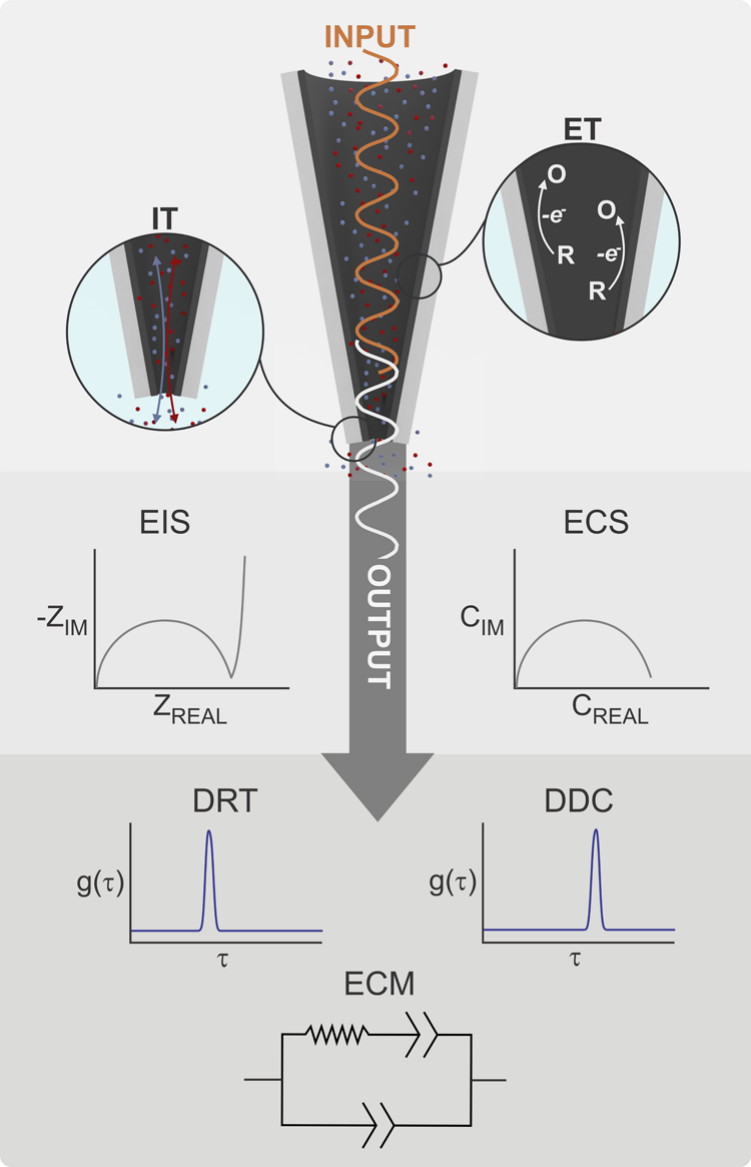
Schematic representation of the electrochemical techniques used in this work and the complementary strategies applied to obtain quantitative information. R and O correspond to the reduced and oxidized forms of the redox probe. Input corresponds to the perturbation, output refers to the data collected from the instrument, *Z*: impedance. *C*: capacitance. *g*(*t*): distribution function. τ: time, REAL and IM subindexes correspond to real and imaginary components, IT: ion transport, ET: electron transfer, EIS: electrochemical impedance spectroscopy, ECS: electrochemical capacitance spectroscopy, DRT: distribution of relaxation time, DDC: distribution of differential capacitance, ECM: electrical equivalent circuit model. For simplicity, the solution inside the CNP was not represented. However, in all cases, the experiments were performed using open CNPs, with the tip partially filled with solution due to capillary effects.

## Materials and Methods

### Materials

Potassium chloride KCl (99.5%), bovine serum albumin BSA (98%), and K_4_Fe^[II]^(CN)_6_·3H_2_O (99%) were purchased from VWR chemicals. Ag wires (0.5 mm diameter), iron chloride FeCl_3_ (97%), and sodium hypochlorite solution NaClO (6–14% chlorine) were obtained from Merck. Quartz capillary tubes without filament (0.7 mm inner diameter, 1.0 outer diameter, and 10 cm length) were provided by Sutter Instrument (Novato, CA). All the reagents were employed as received. All the solutions were prepared in ultrapure water (18.2 MΩ cm at 25 °C, Milli-Q water system, Merck Millipore).

### Carbon Nanopipette Fabrication and Modification

Nanopipettes were fabricated by pulling quartz capillary tubes with a CO_2_ laser-based puller P-2000 from Sutter Instrument. The program employed was the following: Heat 700, Filament 4, Velocity 60, Delay 145, and Pull 175. As a result, nanopipettes with tip radii around 60 nm were obtained (see field-effect scanning electron microscopy analysis in Section S1, Figure S1, SI file). After that, the inner surface of the glass nanopipettes was modified with a carbon layer via chemical vapor deposition.^29^ For this, the glass nanopipettes were exposed to a mixture of 0.2 L min^–1^ methane and 0.6 L min^–1^ argon at 925 °C for 3.5 min.^6^ The CNP was modified with BSA protein by immersing the pipet in a 5 mg/mL pH 6 protein solution for 1 h. Subsequently, CNP was rinsed with deionized water and cleaned by immersing it in the measurement solution (0.75 mM ferrocyanide in 0.3 M KCl) for 2 h.

### Electrochemical Setup

The electrochemical cell consisted of a three-electrode system composed of an Ag/AgCl wire as the reference electrode (RE), a Pt rod (1.92 mm diameter, ∼2 cm^2^, Metrohm Nordic AB) as the counter electrode (CE), and the CNP as the working electrode (WE). The Ag/AgCl wire was prepared by soaking a 5 cm length of Ag wire (0.5 mm diameter) into a diluted NaClO solution (0.6–1.4% chlorine active) for 2 h. The Ag wire (0.5 mm diameter, ∼7 cm length) was inserted into the back of the capillary tube to contact the carbon layer. The carbon layer inside the quartz capillary provided the needed electrical properties to use the CNP as WE. The extreme of the Ag wire outside the capillary was connected to the potentiostat terminal. During the measurements, WE, CE, and RE were positioned approximately 1 cm from each other. The electrochemical cell was placed inside a Faraday cage (Rittal, GmbH & Co. KG). The potentiostat used in all the experiments was a VIONIC from Metrohm operated with the Intello 1.5 software. Kramers–Kronig analysis and ECM fittings were conducted with the NOVA 2.1.4 software (Metrohm).

CVs were typically recorded at 50 mV s^–1^ in the potential window between −0.2 and 0.6 V, otherwise mentioned. Peak currents *I*_p_ were obtained by first subtracting the background. Voltammetric charge was calculated as the ratio between the voltammetric peak area and the scan rate. Formal potentials *E* ^0′^ were estimated as *E* ^0′^∼ (*E*_p,a_ + *E*_p,c_)/2 where *E*_p,a_ and *E*_p,c_ were the peak potentials for the anodic and cathodic reactions, respectively.

Potentiostatic EIS experiments were performed in the frequency (*f*) window from 10^6^ Hz to 0.1 Hz (10 points per decade), applying a 10-mV amplitude sinusoidal perturbation. To study the system at different voltages, the sinusoidal perturbation was superimposed on a given direct current potential (*E*_DC_). In all the cases, the system was preconditioned at the *E*_DC_ for 10 s before the measurement. After the measurement, the data were analyzed by Kramers–Kronig consistent circuit analysis to check their validity (in all the cases, a χ ∼ 10^–5^ or below was obtained). Once the validity was checked, the EIS results were analyzed by ECM, ECS, DRT, and DDC, as explained below.

ECS analysis was obtained from the EIS data. Then, the impedance raw data was converted to the capacitance domain by employing the following expressions^26^

1 $$C=\frac{1}{j\omega \left|Z\right|}$$

2 $$C_{\mathrm{IM}}=\frac{Z_{\mathrm{REAL}}}{\omega |Z{|}^{2}}$$

3 $$C_{\mathrm{REAL}}=\frac{-Z_{\mathrm{IM}}}{\omega |Z{|}^{2}}$$

where *C* is the capacitance, *Z* is the impedance, *C*_IM_ and *C*_REAL_ are the imaginary and real components of the capacitance, respectively, *Z*_IM_ and *Z*_REAL_ are the imaginary and real components of the impedance, respectively, ω is the angular frequency (ω = 2π*f*) and $j=\sqrt{-1}$ .

In all cases, measurements were performed under stable internal volume conditions. To ensure this, the CNP was immersed in the solution and left until no changes were observed across successive CV scans. The solution inside the internal volume of the CNP was provided by natural capillarity (no external pressures applied) and, for this reason, its magnitude depends not only on physical parameters such as size, taper, and cone angle, but also on experimental conditions, including immersion depth and stirring. Given that volume strongly influences the electrochemical response, all comparisons presented in this study were carried out using the same CNP to ensure consistency. In all the cases, the trends have been validated by repeating the experiments with independent samples.

### DRT and DDC Analysis

DRT method enables the analysis of the relaxation characteristics of a given electrochemical system by solving the Fredholm integral equation^14,16,17^

4 $$Z\left(f\right)=R_{\mathrm{SOL}}+\int_{0}^{\infty }\frac{g_{\mathrm{DRT}}\left(\tau \right)}{1+j2\pi f\tau }d\tau$$

where *Z*(*f*) is the impedance at the frequency *f*, *R*_SOL_ is the solution resistance, *g*_DRT_(τ) is the DRT function, and τ is the relaxation time. Equation 4 can be understood as the sum of an ohmic resistance at *f* → ∞ (*R*_SOL_) and infinite (RC)-elements in series (Voigt circuit).^30^ As the frequency data was recorded in a logarithmic scale with 10 points per decade, *τg*_DRT_(τ) can be replaced by γ_DRT_(ln τ) and eq 4 is rewritten as follows^30^

5 $$Z\left(f\right)=R_{\mathrm{SOL}}+\int_{-\infty }^{\infty }\frac{\gamma_{\mathrm{DRT}}\left(\ln\tau \right)}{1+j2\pi f\tau }\mathrm{dln}\tau$$

The mathematical issue behind eq 5 is ill-posed and requires special methods to be solved. In this work, we employ the open-access DRTtools toolbox for MATLAB software (MATLAB R2023b) developed by Ciucci and co-workers (it is worth mentioning that the same toolbox is freely available for Python).^30^ DRT distributions are obtained from EIS data via the Tikhonov regularization. This method includes a crucial variable, the regularization parameter λ. Small λ values (e.g., <0.001) enhance the DRT resolution. However, excessively decreasing λ may lead to the emergence of artifacts, such as false peaks and oscillations. For this reason, DRT analysis required the study of the optimal λ as explained below in the text.

The DRT method is well applied to EIS data consisting of an RC-element series. This method requires *Z* to be convergent in the limit of low frequencies. This is not the case for the present study; therefore, the analysis could not be conducted at low frequencies. To address this limitation, DDC was then applied. This method involves the same procedure as DRT, but the EIS spectrum is converted to the convergent ECS spectrum before the deconvolution (see eqs 1–3).^3,21,31^ Thus, in analogy to eq 5

6 $$C\left(f\right)=\int_{0}^{\infty }\frac{\gamma_{\mathrm{DDC}}\left(\ln\tau \right)}{1+j2\pi f\tau }\mathrm{dln}\tau$$

where *C*(*f*) is the capacitance at *f* and γ_DDC_(ln τ) is the DDC function. In contrast to the DRT case, eq 6 can be understood as the sum of infinite [RC]-elements in parallel.^31^ Considering the analogy between DRT and DDC, this method was indeed solved by employing the same procedure and software as DRT (DRTtools toolbox for MATLAB^30^).

## Results and Discussion

### EIS Response outside of the Faradaic Window

As shown in Section S2, Figure S2a,b, EIS measurements at open-circuit conditions in a glassy carbon macroelectrode immersed in a 0.3 M KCl solution yield a Nyquist plot characterized by only a straight line across the entire frequency range. Under these conditions, the impedance magnitude is solely governed by the solution resistance and the double-layer capacitance. However, a semicircle appears in the Nyquist plot for the measurement conducted in the same macroelectrode at open circuit potential but in a solution containing equimolar concentrations of both forms of the redox couple (Fe­(CN)_6_^4–^/Fe­(CN)_6_^3–^) (Figure S2a,b).

In contrast to the glassy carbon electrode, the Nyquist plot obtained for a CNP immersed in a 0.3 M KCl aqueous solution without the presence of any redox probe is characterized by two clear features: (i) a semicircle at high/moderate frequencies, and (ii) a vertical line almost parallel to the *y*-axis at low frequencies (Section S3, Figure S3a,b). Qualitatively, the key difference between the macro- and nanoelectrode is the presence of the semicircle, which is attributed to the ion transport phenomenon through the CNP orifice. On the other hand, the vertical line observed at intermediate to low frequencies is attributed to an electrical double-layer charging (*C*_DL_) due to the carbon layer polarization. In all the cases, the negligible changes in the spectra recorded at different direct current voltages (*E*_DC_) from 0 to 0.4 V demonstrated that *R*_CNP_ and *C*_DL_ for the pipet are independent of the bias voltage.

Nyquist plots obtained from ion transport experiments using glass nanopipettes are also characterized by a semicircle at moderate frequencies.^32,33^ To reach such observations, the setup consisted of a glass nanopipette filled with a supporting electrolyte, as well as two Ag/AgCl electrodes placed inside and outside the capillary, respectively. The voltage is applied between the two Ag/AgCl electrodes, which establishes an ion flux across the nanotip. Concomitantly, the resulting semicircle in the Nyquist plot is entirely determined by the ion transport and the characteristics of the pipet surface (purely iontronic contribution). Moreover, since the glass nanopipette is nonconductive and its surface cannot be polarized by an external field, the typical vertical line of thin-layer regimes at low frequencies associated with capacitive or redox processes is not observed.^11,34,35^ Accordingly, the EIS response lacks any electronic contribution from either the charging current (electrical double layer) or redox reactions, in contrast to the main case herein studied based on conductive nanopipettes. It is worth noting that experiments performed with glass nanopipettes at moderate to low electrolyte concentrations have reported an inductive loop at low frequencies, associated with interactions between ions and surface charges.^33,36^ In contrast, the absence of such loops in the CNP experiments shown here is likely related to the relatively high supporting electrolyte concentration, leading to diminished ion selectivity. The ability to extract quantitative information from both iontronic and electronic signals using the same measurement and electrochemical setup represents one of the central aims of this paper. Notably, this dual analysis capability (and the different methods applied) is one of the aspects that distinguishes the present work from previously reported studies on insulating nanofluidic devices, where only ion transport phenomena are assessed.

For CNPs, the presence of a redox probe in the sample solution conditions the selection of the *E*_DC_ value, making this aspect important. Figure 2a–c present the Nyquist and Bode plots obtained for EIS measurements conducted in a solution of 0.10 mM ferrocyanide in 0.3 M KCl at different *E*_DC_ values. When *E*_DC_ was far from the formal potential of the redox couple *E* ^0′^ (e.g., *E*_DC_ = *E* ^0′^ ± 0.2 V), being therefore outside the faradaic window, the EIS responses remained similar to those obtained in the absence of a redox probe. Interestingly, the magnitude of the applied *E*_DC_ became less significant when the voltage lies outside the faradaic window. For instance, |*Z*| varied by less than 15% across the entire frequency range when comparing the EIS responses at *E*_DC_ 0 and 0.4 V. This occurred because the redox probe has a negligible influence on the electrochemical response when the applied bias voltage is not sufficient for triggering oxidation or reduction processes.

**Figure 2:**
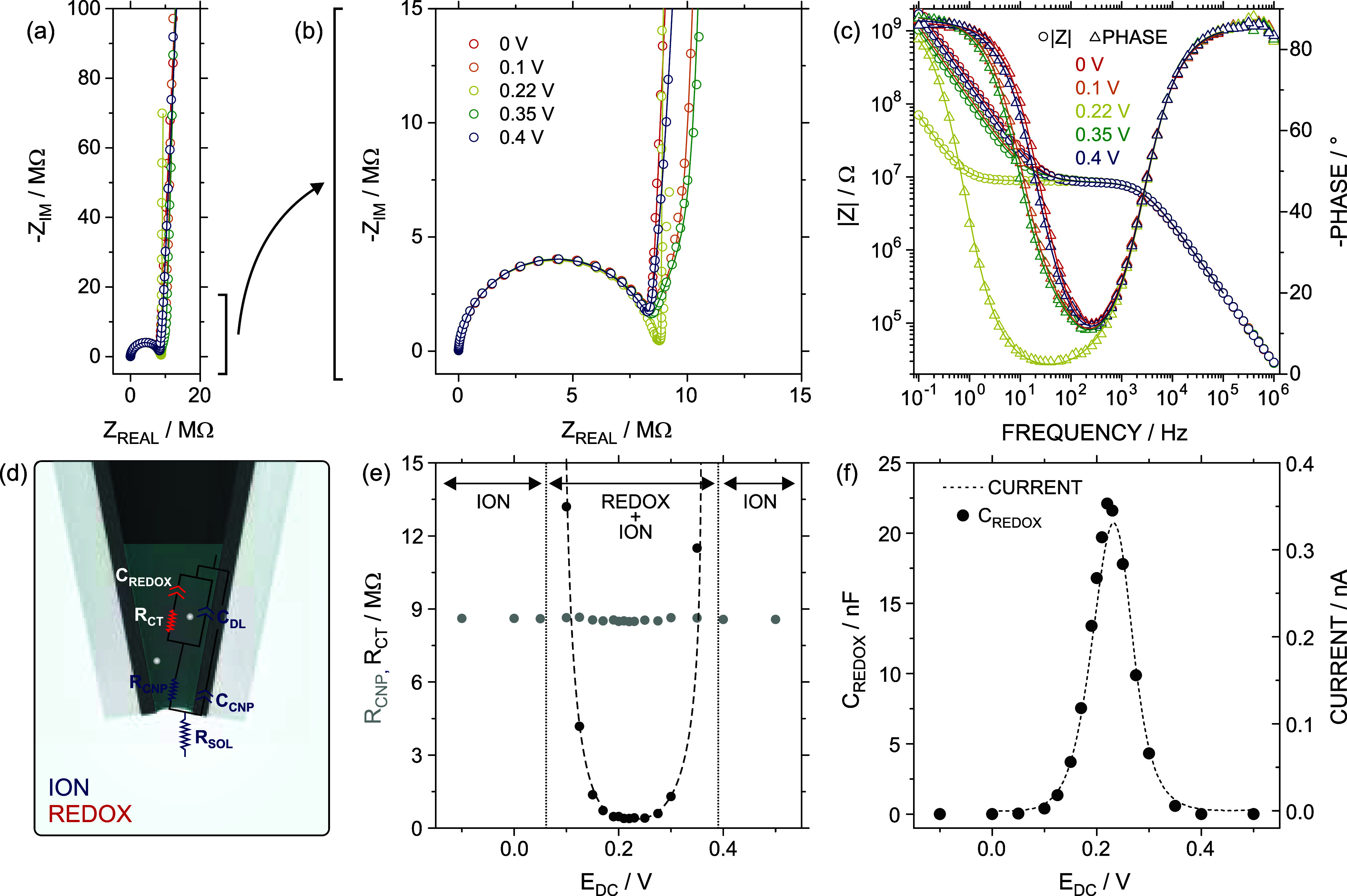
EIS at different *E*_DC_: (a) Nyquist plot and (b) zoom-in. (c) Bode plot. Void circles (and triangles) and lines correspond to the experimental measurement and fitting in terms of ECM, respectively. (d) Scheme of the CNP filled with a certain volume with the proposed ECM. (e) *R*_CNP_ (gray) and *R*_CT_ (black) obtained from ECM fitting at the different *E*_DC_. The dashed line was included as a visual guide. (f) *C*_REDOX_ (circles) obtained from ECM fitting at the different *E*_DC_. The current of the forward scan in the CV at 10 mV s^–1^ (dashed line) was also included to demonstrate the good agreement between both kinds of measurements. All the measurements were conducted in 0.10 mM ferrocyanide +0.3 M KCl.

Analogous analysis of EIS results can be performed in terms of the Bode plots (|*Z*| and phase angle vs frequency). In the cases of absence and presence of a redox probe but with the *E*_DC_ being outside the faradaic window, the Bode plots remained unchanged across different *E*_DC_ values (Figure 2c). However, it is valuable to analyze the following distinctive features. At frequencies above 100 kHz, the phase angle approached 90°, primarily due to the capacitance of the CNP *C*_CNP_. Around 250 Hz, the phase angle reached a minimum of 10°, reflecting a transition from capacitive to resistive behavior caused by ion transport through the CNP, *R*_CNP_. At frequencies below 10 Hz, the phase angle again tended to 90°, driven by the double-layer capacitance of the carbon layer, *C*_DL_.

Similarly, the |*Z*| vs frequency plot denotes three different regions: (a) a high-frequency capacitive region (>1000 Hz) attributed to *C*_CNP_, where |*Z*| increased as the frequency diminished; (b) a resistive region at moderate frequencies, dominated by *R*_CNP_ where |*Z*| exhibited frequency-independent behavior; and (c) another capacitive region (<10 Hz) governed by *C*_DL_, where |*Z*| again increased as the frequency diminished. These three regions delineate two breaking points where the phase angle equals 45°. These breaking points correspond in turn to characteristic times of the underlying processes and are very sensitive to experimental conditions (CNP properties, supporting electrolyte concentration and composition, redox probe concentration, and pipet volume).^11^

### EIS Response inside the Faradaic Window

When *E*_DC_ approaches *E* ^0′^ (i.e., within the faradaic window), the alternating voltage superimposed on the direct current enables the oxidation and reduction of the redox probe. Consequently, electron transfer begins to contribute to the overall response. The Nyquist plot at high/moderate frequencies resembled that of the EIS response for *E*_DC_ values outside the faradaic window, with some slight variations at moderate/low frequencies (Figure 2a,b). Notably, more evident changes were found in the corresponding Bode plots (Figure 2c). The frequency at which the capacitive behavior emerged within the low-frequency region was found to decrease as *E*_DC_ approached to *E* ^0′^. This resulted in an expanded frequency-independent domain in the |*Z*| plot and *a* broader minimum in the phase angle plot. Also, the second breaking point shifted to lower frequencies (from 25 to 0.8 Hz). The appearance of the capacitive behavior at low frequency in the experiment conducted in the presence of a redox probe at *E*_DC_ ∼ *E* ^0′^ indicates that the electrical potential can propagate throughout the entire volume of the CNP within the scanned frequency range, reaching the system’s charge storage limit. This is a hallmark of thin-layer systems. The presence of a thin-layer behavior was further evidenced by analyzing the CVs at different scan rates, as shown Section 4, Figure S4. Consequently, this low-frequency capacitive behavior, known as charge saturation region, exhibits a capacitance magnitude (*C*_REDOX_) that encodes quantitative information on the total population of redox-active species present within the nanofluidic architecture.^35^

### ECM Analysis

One of the most common methods for obtaining quantitative information from EIS measurements consists of using ECMs: EIS results are modeled using an electrical circuit where each component is correlated to a physicochemical feature of the system. To conceive the ECM, certain aspects were considered. For purely iontronic signals, nanofluidic devices have been effectively modeled using a simplified Randles circuit, R­(R||C).^37–39^ This circuit is set out with a resistance related to bulk solution conductivity (*R*_SOL_) in series with the parallel combination of *R*_CNP_ and *C*_CNP_. For CNPs where an external potential is applied to the carbon layer, an additional (RC) component composed of a *C*_DL_ and *R*_CT_ is added in series to the *R*_CNP_. In this, *R*_CT_ represents the electron transfer resistance. Then, to account for the thin-layer electrochemical behavior, an additional capacitance (*C*_REDOX_) is placed in series with *R*_CT_ to represent the total charge accumulated due to the redox reaction. This component has previously been employed for electrodes with adsorbed redox centers, which represents a similar example to redox reactions in thin-layer domains.^35^ As a result, the proposed circuit for the system is *R*_SOL_([*R*_CNP_([*R*_CT_ *C*_REDOX_]*C*_DL_)]*C*_CNP_) (Figure 2d). This circuit integrates elements from both nanofluidic device models and thin-layer electrochemical behavior.

It was found that, the fitting to the proposed ECM satisfactorily predicts the experimental trend of the EIS results obtained at *E*_DC_ ≫ 0.2 V and *E*_DC_ ≪ 0.2 V, with a goodness-of-fit parameter chi-squared χ < 0.005 (lines in Figure 2a–c). To further improve the fitting quality, all capacitive components were replaced with constant phase elements (CPEs), being this a common approach to account for nonidealities such as carbon porosity and surface inhomogeneity.^2^ Moreover, CPEs are defined by two characteristic parameters, the CPE constant (*Y*_0_ *)* and the exponent (*n*). In particular, *n* takes values from 0 to 1 that accounts for the deviation of the CPE from the ideal capacitor. When *n* → 1, the CPE behaves as an ideal capacitor and *Y*_0_ is the capacitance (*C*). Considering that in all cases the CPE exponents (*n*) were very close to the ideal capacitor (*n* > 0.96), *Y*_0_ values were directly referred to as capacitances in the text (e.g., C_DL_ instead of CPE_DL_). As shown in Table S1, outside the faradaic window (e.g., −0.1, 0, 0.05, and 0.5 V), *R*_CT_ values reached tens of MΩ, and *C*_REDOX_ represented less than 10% of the *C*_DL_. Under these conditions, the contribution of the redox reaction to the EIS response is negligible, allowing the circuit to be simplified to *R*_SOL_([*R*_CNP_ *C*_DL_]*C*_CNP_) without any significant variation compared to the complete model (<5%). From the ECM analysis of the EIS data at −0.1, 0, 0.05, and 0.5 V, *R*_CNP_, *C*_CNP,_ and *C*_DL_ were estimated as 8.59 ± 0.02 MΩ, 10.7 ± 0.3 pF, and 0.86 ± 0.05 nF (average ± standard deviation, *n* = 4), respectively. These results confirmed that *C*_CNP_ and *R*_CNP_ are independent of the applied *E*_DC_ (Figure 2e).

Within the faradaic window (0.05 V < *E*_DC_ < 0.35 V), the proposed circuit also replicates the experimental EIS trends, with fitting parameters χ around 0.006 in all the cases. The fitting quality decreased slightly compared to conditions outside the faradaic window, particularly in the transition from the semicircle to the charge saturation region. Influences from redox probe mass transport or adsorption were excluded to simplify the model. The results provided in Figure 2e,f revealed that *C*_REDOX_ and *R*_CT_ were highly sensitive to the applied bias voltage. This is because the *E*_DC_ magnitude determines the redox probe concentrations, and the faradaic contribution to EIS measurements sensitively depends on the ratio between the oxidized and reduced species. This aspect is comprehensively addressed in Section S5, SI file.

The analysis of EIS in terms of ECM enabled different conclusions about the system. Overall, the electrochemical response highly depends on the ion transport and redox reaction. In contrast to other techniques, such as CV, EIS results at *E*_DC_ = 0 V (*E*_DC_ ≪ *E* ^0′^) made it possible to determine the tip resistance by fitting the response to an *R*(*R*||*C*)*C* model. Also, information about the redox reaction was mainly obtained by analyzing EIS results *E*_DC_ = *E* ^0′^. In this case, the major complexity of the system and the addition of new circuit components made it more complex to fit the experimental results. However, obtaining important parameters such as *R*_CT_ and *C*_REDOX_ was still possible. For this reason, EIS measurements were always evaluated inside and outside the faradaic window.

Several equivalent circuits can adequately describe the EIS results.^13^ A useful method to eliminate an unsuitable ECM involves varying a specific experimental condition to observe if it produces the expected changes in the circuit parameters. To further validate the proposed equivalent circuit, additional experiments were conducted under varying KCl concentrations (different *R*_CNP_) and using FeCl_3_ as the redox probe; a compound with slower kinetics (higher *R*_CT_) compared to ferrocyanide. Figures S5 and S6 present EIS results obtained at different KCl concentrations and employing FeCl_3_ as the redox probe.^9^ Variations in KCl concentration produced significant changes in the diameter of the EIS semicircle and the ECM fitting revealed notable changes in *R*_CNP_ (Figure S5, and Table S2). Additionally, the presence of FeCl_3_ led to the appearance of a second semicircle at lower frequencies when *E*_DC_ was set within the faradaic window (Figure S6). Moreover, the change in the EIS response was reflected as an increase in *R*_CT_ within the ECM, due to the irreversible electrochemical behavior of FeCl_3_. Therefore, in both scenarios, the proposed equivalent circuit successfully fitted the experimental data and provided a coherent interpretation of the variations observed in the EIS results, reinforcing the model’s validity.

### Electrochemical Capacitance Spectroscopy (ECS)

In EIS, the information related to the number of moles reacting on the electrode surface mostly lies in the capacitive behavior of the charge saturation region, *C*_REDOX_. In particular, at *E*_DC_ = *E* ^0′^ to the following expression (see Section S5, SI file, for further details)

7 $$C_{\mathrm{REDOX}}=\frac{F^{2}Vc^{*}}{4\mathrm{RT}}$$

where c* corresponds to the total redox probe concentration (i.e., c* = *c*_O_ + *c*_R_) inside the CNP. Notably, the linear relationship of *C*_REDOX_ and c* motivates the use of ECS as the analysis method for sensing purposes.

ECS results were graphically represented in a similar way as those obtained in EIS. Specifically, Figure 3a shows the transformation to the capacitive plane of the EIS results exhibited in Figure 2a. The *C*_IM_ vs *C*_REAL_ plot (analog to the Nyquist plot) obtained with the CNP immersed in 0.10 mM ferrocyanide +0.3 M KCl was characterized by a well-defined semicircle with a diameter that sensitively depended on *E*_DC_. For reference, in macroelectrodes, this profile is achieved when the electrode surface is modified with a thin redox-active film.^40^ Moreover, the ECS Nyquist plot obtained for a GC electrode in a ferrocyanide solution resulted in a different behavior from the one displayed in CNP due to the diffusion-controlled regime at low frequencies (Section S6, Figure S7).

**Figure 3:**
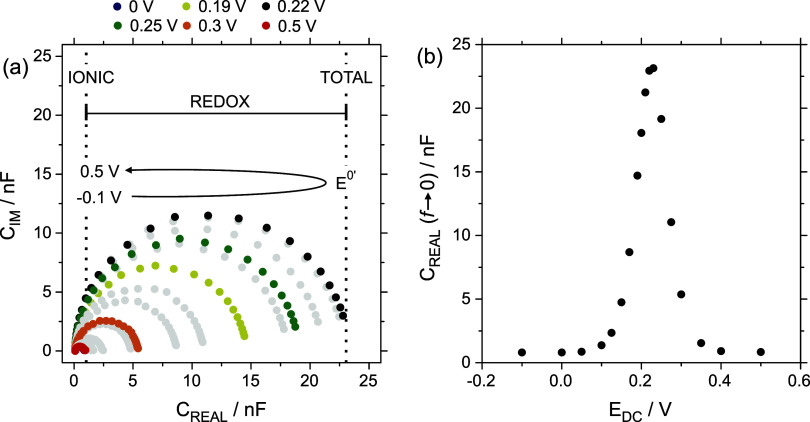
(a) ECS Nyquist plot at different *E*_DC_ ranging from −0.1 to 0.5 V. Measurements at 0, 0.19, 0.22, 0.25, 0.3, and 0.5 V are represented in different colors to evidence trends in the results. (b) *C*_REAL_ (*f* → 0) in terms of *E*_DC_. All the measurements were conducted in 0.3 M KCl and 0.10 mM ferrocyanide. In all the cases, the plots obtained at 0 and 0.5 V overlap (further details are available in the SI).

Considering the range of frequencies evaluated during the EIS experiment, the CNP’s total capacitance is determined by two main contributions: the electrical double layer and the redox reaction. The first is usually called non-Faradaic capacitance, while the latter is the redox capacitance, *C*_REDOX_ (pseudocapacitance). The contribution of the redox reaction to the ECS spectra obtained at an *E*_DC_ outside the faradaic window was negligible, and therefore, the diameter of the semicircle (i.e., *C*_REAL_ (*f* → 0)) can be approximated to the nonfaradaic or ionic capacitance. Then, outside the faradaic window, the EIS and ECS responses remained invariant at the different *E*_DC_ values, as shown in Figure S8. The total capacitance obtained in the ECS outside the faradaic window (OF) (C_REAL_^OF^ (*f* → 0)) can be approximated to *C*_DL,_ since the other nonfaradaic capacitive component of the system (i.e., *C*_CNP_) can be assumed as negligible compared to *C*_DL_ (∼80 times smaller, see Table S1). Conversely, when *E*_DC_ took values inside the faradaic window, *C*_REDOX_ began to contribute to the CNP total capacitance. Thus, *C*_REAL_ (*f* → 0) (i.e., the total capacitance) increased as *E*_DC_ approached *E* ^0′^ due to the increment in *C*_REDOX_.

The plot of *C*_REAL_ (*f* → 0) vs *E*_DC_ (Figure 3b) exhibited a maximum value when *E*_DC_ ∼ *E* ^0′^. Advantageously, the subtraction of the ionic component (*C*_REAL_^OF^ (*f* → 0)) from the total capacitance obtained in the ECS recorded inside the faradaic window (IF) (*C*_REAL_^IF^ (*f* → 0)) enabled the decoupling of *C*_REDOX_ from *C*_DL_

8 $$C_{\mathrm{REDOX}}=C_{\mathrm{REAL}}^{\mathrm{IF}}\left(f\to 0\right)-C_{\mathrm{REAL}}^{\mathrm{OF}}\left(f\to 0\right)\approx C_{\mathrm{REAL}}^{\mathrm{IF}}\left(f\to 0\right)-C_{\mathrm{DL}}$$

*C*_REAL_^IF^ (*f* → 0) and *C*_REAL_^OF^ (*f* → 0) were determined as the semicircle diameter in the *C*_IM_ vs *C*_REAL_ plot. C_REAL_^OF^ (*f* → 0) at 0 V (i.e., ∼*C*_DL_) and *C*_REDOX_ at *E*_DC_ ∼ *E* ^0′^ were 0.83 and 22.22 nF, respectively, with a good agreement with the values obtained by ECM (*C*_DL_ = 0.81 nF and *C*_REDOX_ = 22.00 nF): differences lower than 5%. However, the ECS analyses involved a much more straightforward alternative for the cases where only the capacitance values are needed, because it only requires the determination of the semicircle diameter instead of the ECM modeling.

Similar quantitative analysis can be performed by plotting *C*_REAL_ vs *f*, as shown in Figure S8b. Also, another manner to represent the ECS results is a *C*_IM_ vs *f* plot, which provides information on the characteristic times τ (τ = 1/2π*f*_max_) of the capacitive phenomena involved in the system (Figures S8c and S9). These results were comprehensively addressed in Section S7, SI file.

### Identification of Characteristic Times

ECM analysis can be challenging when multiple processes share similar relaxation frequencies, and different circuits can often fit the same EIS data. In contrast, the DRT method requires no prior assumptions and provides quantitative insights into the characteristic times and resistances of the processes contributing to the signal, which justify exploring the potential of the application of DRT analysis to nanofluidic devices. Figure 4a presents the DRT spectra in terms of τ, while Figure 4b,c depict the experimental data (circles) and the fitting (lines) obtained from the DRT model (same EIS data as those presented in Figure 2). In all the cases, a λ of 0.001 was selected for an initial analysis, which is a standard value validated in the literature.^17^ Notably, the mathematical resolution behind the DRT analysis strongly depends on the regularization parameter λ, and therefore, it was carefully selected at the beginning of the treatment. Overall, decreasing λ enabled the increment of the resolution but also led to the appearance of artifact peaks. Indeed, the diminution of λ to 0.001 significantly improved the fitting of the experimental data (comparison of DRT plots obtained at different λ is available in Section S8, Figure S10). Further decrease of this value did not produce significant variations in the EIS fitting and resulted in the appearance of false peaks in the DRT spectrum. Conversely, the increment of λ up to values around 0.01–0.1 led to a significant broadening in the DRT peak (which could contribute to a loss of resolution) with a notable deterioration in the fitting of the experimental data.

**Figure 4:**
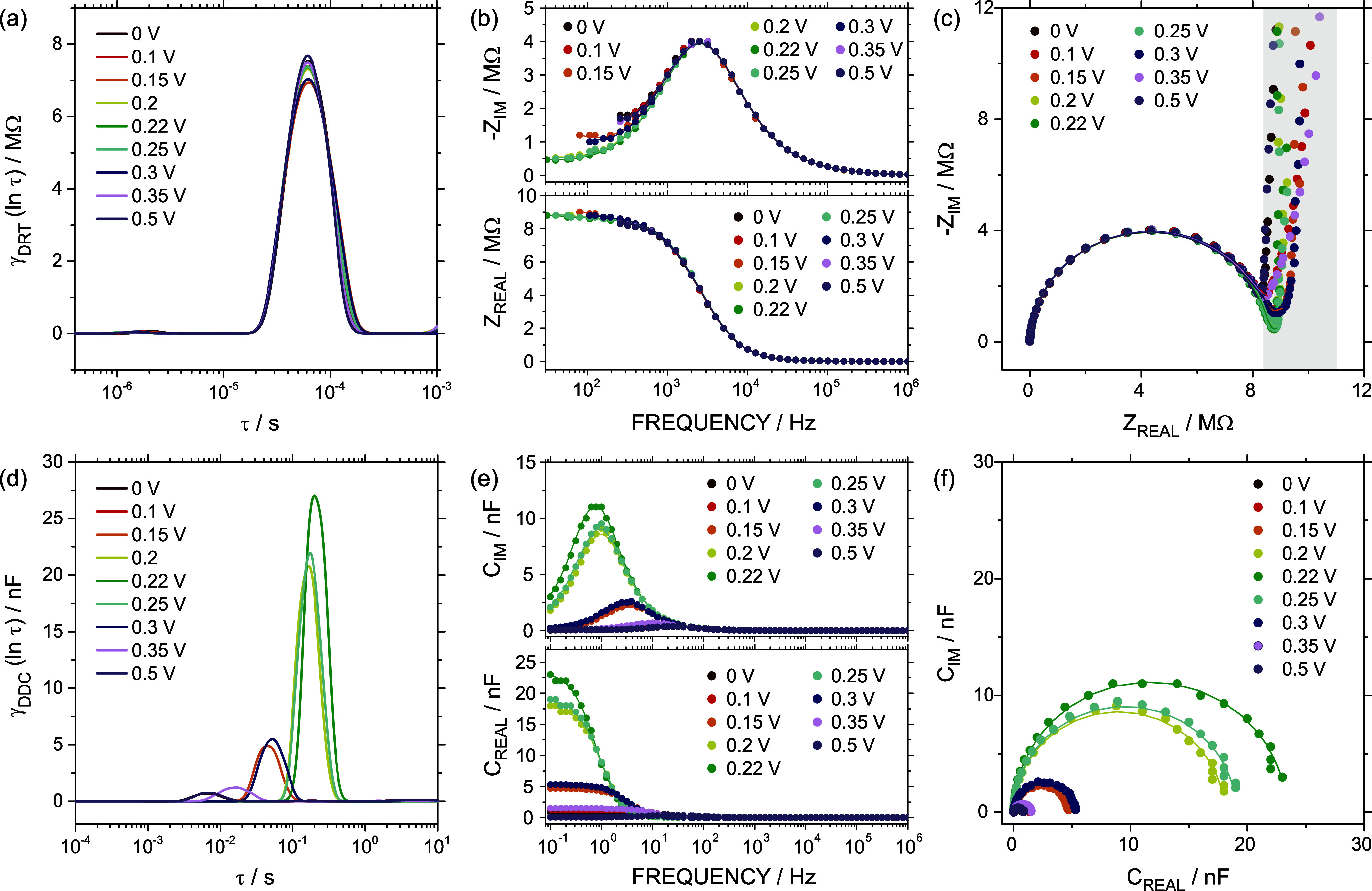
(a) DRT spectra in terms of τ. (b) −*Z*_IM_ (top), *Z*_REAL_ (bottom), and (c) EIS Nyquist plot in terms of the frequency for EIS recorded at different *E*_DC_ voltages. The circles and lines represent experimental EIS data and fitting results obtained from DRT, respectively. The gray region was not considered in the DRT analysis. (d) DDC spectra in terms of τ. (e) *C*_IM_ (top), *C*_REAL_ (bottom), and (f) ECS Nyquist plot in terms of the frequency for ECS recorded at different *E*_DC_ voltages. The circles and lines represent experimental ECS data and fitting results obtained from DDC, respectively. All the experiments were performed in 0.3 M KCl and 0.1 mM ferrocyanide.

DRT plots in the range 10^–3^ s > τ > 10^–6^ s in Figure 4a primarily revealed one peak at 61.6 ± 0.5 μs (2580 ± 0.03 Hz) at the different *E*_DC_ values that were tested. This feature agrees with the behavior observed in *Z*_IM_ vs *f* plot (Figure 4b), where the peak position remained almost constant at the different *E*_DC_ values with a frequency position around 2500 Hz (similar to the value obtained for the first breaking point in Figure 2c). When the time window is extended to τ = 10^–1^ s, a second peak was evidenced. This peak shifted toward longer times as *E*_DC_ approached *E* ^0′^ (Figure S11). This component was due to the presence of the charge saturation region (redox contribution), as demonstrated by the capacitive tail in the Nyquist plot (Figure 4c). However, DRT-based results are applicable across the entire frequency range only in the case of systems that behave as Voigt circuits (a series of RC elements). Considering that, due to the thin-layer properties of the device, *Z*_IM_ → ∞ when *f* → 0 Hz (high τ values), DRT analysis provided meaningless peaks at τ > 10^–3^ s. For this reason, the analysis was limited to the frequency region where the ion transport is the main contribution (10^3^–10^6^ Hz or 10^–6^–10^–3^ s).

Beyond the calculation of characteristic times, another advantage of DRT is that it allows obtaining quantitative information about the system’s resistance without requiring precise details of the ECM. This is achieved by integrating the peak obtained in the *g*(τ) vs τ plot (see eq 4), with the resulting area providing insights into the polarization resistance of the process. In the case analyzed here, the peak area remains relatively constant across different *E*_DC_, yielding a value of 8.4 ± 0.1 MΩ. This represents a difference of only 2% compared to the *R*_CNP_ value obtained by the ECM model (8.59 ± 0.2 MΩ).

The DDC approach has been proposed as an alternative to obtain information on characteristic times in systems exhibiting a low-frequency capacitive behavior.^18^ As shown in Figure 4d, the DDC spectra presented a single peak that shifted toward lower frequencies as *E*_DC_ → *E* ^0′^. The position of the peak was consistent with that observed in the *C*_IM_ vs *f* plot (Figure 4e). Similarly to the DRT case, the prediction of the EIS behavior from the DDC result accurately agrees with the experimental results (lines in Figure 4e,4f). In this case, the peak integration of *g*_DDC_ in terms of τ led to a value of *C*_DL_ = 0.77 nF ± 0.02 nF (average ± standard deviation of the results at *E*_DC_ = −0.1, 0, 0.05, and 0.5 V) and *C*_REDOX_ (at *E*_DC_ = *E* ^0′^) of 22.5 nF, which represent variations around 5% regarding the values obtained by ECM. At this point, we would like to emphasize that the ability to obtain *C*_REDOX_ straightforwardly could enhance the potential of DDC in the (bio)­sensing field, particularly for sensors utilizing ECS analysis. This conclusion extends beyond the CNP case because it could apply to macroelectrode-based capacitive sensors.^27^ Thus, even in macroelectrodes, DDC can serve as a simple tool to determine the differences in the capacitance promoted by the presence of an analyte without the necessity of any assumptions or an electrical equivalent circuit.

Overall, these results demonstrate that, beyond the characteristic times, DRT and DDC involve simple methods to quantify important parameters such as *R*_CNP_, *C*_DL,_ and *C*_REDOX_. However, for a reversible redox probe, deconvolving ion resistance (*R*_CNP_) and electron transfer resistance (*R*_CT_) at a given *E*_DC_ was challenging because *R*_CT_ ≪ *R*_CNP_. To gain further insight into this issue, DRT and DDC analysis for an irreversible probe (FeCl_3_) where *R*_CT_ > *R*_CNP_ was also analyzed (Figure S12). In this case, ion and redox contributions are separated in the EIS and ECS spectrum, which translates into two discernible peaks in the DRT and DDC results. Thus, the resistance and capacitance values for each process can be estimated in the same plot, leading to magnitudes of 5.7 MΩ, 143 MΩ, 0.21 nF and 1.92 nF for *R*_CNP_, *R*_CT_, *C*_DL,_ and *C*_REDOX_, respectively. Except for *R*_CT_, where differences were around 20%, the results obtained from DRT, DDC, and ECM were similar (<10% of difference).

### Investigation on the Effects of the Redox Probe Concentration on the Electrochemical Response

To explore the EIS potential providing quantitative information, EIS routines were evaluated at *E*_DC_ = 0 V and *E* ^0′^ while varying the concentration of the redox probe redox probe concentrations in the 35–750 μM range. Then, the data were fitted to the ECM (Figure S13). The results demonstrated a decrease in *R*_CT_ and an increase in *C*_REDOX_ as the redox probe concentration increased. The increment in *C*_REDOX_ followed a rather linear trend with the concentration, whereas *R*_CT_ linearly decayed with the inverse of the concentration. While these relationships followed the tendencies predicted by the thin-layer theory, it is worth mentioning that, especially at low concentrations, the fitting can provide high uncertainties in the determination of *R*_CT_ and *C*_REDOX_. This is explained by the low contribution of the redox reaction to the EIS signal (see below).

Overall, these results suggest that the analysis in terms of ECM is reliable for the determination of *R*_CT_ and *C*_REDOX_ in cases where the concentration of the redox probe is significantly high and hence *C*_REDOX_ is comparable or higher than *C*_DL_ and there are clear differences in the EIS responses at *E*_DC_ ≫ *E* ^0′^ (or *E*_DC_ ≪ *E* ^0′^) and *E*_DC_ = *E* ^0′^. In this context, the technique could be an interesting alternative to obtain fundamental information on redox reactions under nanoconfinement. However, when the probe concentration is low and the redox reaction contributes minimally to the EIS response, fitting the data to collect quantitative information about the reaction becomes challenging. This issue, together with the ambiguity that multiple equivalent circuits can provide acceptable fittings, undoubtedly represents a clear disadvantage of this method, especially when the technique is employed for analytical purposes.

ECS analysis did not show significant changes in the total capacitances at *E*_DC_ = 0 V varying the redox probe concentration (Figure S14a). Conversely, there was an increment in the semicircle diameter of the ECS obtained at *E*_DC_ = *E* ^0′^ as the redox probe concentration increased (Figure 5a). Thus, while the variation in the concentration did not produce any effect on *C*_DL_, it led to an increment in *C*_REDOX_, which reinforces the trend obtained by ECM analysis. Furthermore, the analysis of *C*_IM_ in terms of *f* demonstrated that the increment in the total capacitance is accompanied by an increase in the characteristic time (decrease of the frequency) τ_REDOX_ of the thin-layer redox reaction (Figure S14b). This fact is indeed expected when considering that more time for the total consumption of the redox probe is required as the number of moles inside the CNP increases. For this reason, both *C*_REDOX_ and τ also sensitively depend on the volume inside the CNP played a central role. The volume inside the CNP determines the time scale required in the experiment to obtain a total conversion and sensitivity.^11^ Higher volumes inside the CNP typically increase the signal-to-noise ratio (the peak current or charge associated with a given analyte concentration); therefore, to use open CNPs as analytical platforms, the optimization and accurate control of the volume is of paramount importance.

**Figure 5:**
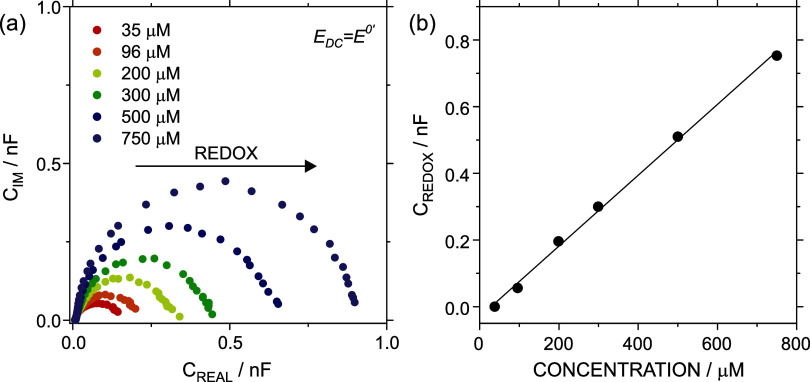
(a) ECS Nyquist plots at *E*_DC_ = *E* ^0′^ (inside faradaic window) for increasing ferrocyanide concentrations. (b) *C*_REDOX_ in terms of ferrocyanide concentration. All the measurements were conducted in 0.3 M KCl as the supporting electrolyte.

The analysis of *C*_REDOX_ in terms of the concentration followed a linear relationship (*r* ^2^ = 0.997) with a slope of 1.06 × 10^–3^ (±2 × 10^–5^) nF/μM (Figure 5b). Notably, like CV analysis where the voltammetric charge can be related to the pipet volume by applying the Faraday law, eq 7 can be used to determine the volume inside the CNP. The value estimated by the observed ECS data was 1.12 ± 0.02 pL, which represented a discrepancy lower than 10% from those obtained by applying the Faraday law with the voltammetric charge obtained in the CV routines (1.04 ± 0.02 pL). Correspondingly, it is also possible to obtain the total number of moles (VC*) inside the CNP by ECS (EIS) measurements. For the cases where the volume is a known parameter, this result implies that ECS could be employed for calibration-free sensing, with the direct calculation of the concentration from the electrochemical charge by accurately knowing the active sample volume inside the CNP.

While the application of ECS simplifies the determination of *C*_REDOX,_ which could be useful for analytical purposes, this is at the expense of obtaining information on the system resistance. Accordingly, somehow, ECS analysis is complementary to EIS. However, compared to traditional CV, the use of ECS did not demonstrate significant improvement of the analytical performance, as reveals the analysis in Figure S15. Conversely, both approaches yielded similar results, and considering the higher simplicity of experimental acquisition and data treatment, this result seems to suggest that CV may be more suitable for this type of quantitative analysis.

### Impact of the Presence of Nonredox Active Species on the Electrochemical Response

When a redox compound undergoes oxidation or reduction at the pipet surface, EIS or ECS can provide information comparable to that obtained from thin-layer CV experiments (e.g., the volume inside the CNP). However, for quantitative analytical studies, the greater complexity associated with signal acquisition makes CV a more favorable alternative. Importantly, EIS has been widely employed in macro-electrodes to evidence recognition events involving nonredox moieties as well as to demonstrate the successful modification of electrode surfaces. Notably, the modification of the substrate is a key step in the development of sensors because it enables the improvement of important properties such as selectivity and sensitivity. Considering the peculiarities of the EIS response of CNP, it is valuable to gain insights into the main features of the response for the case where a nonredox active building block is immobilized onto the CNP walls and compare them to those obtained by the traditional CV. In this context, the EIS and CV responses of ferrocyanide were studied before and after functionalization of the CNP internal walls with bovine serum albumin (BSA) protein (66 kDa). For that, the CNP was immersed in a solution of 5 mg/mL of the protein for 1 h and washed in the measurement solution for 2 h. Then, we recorded EIS measurements from 10^6^ Hz to 0.1 Hz at *E*_DC_ = 0 V and *E* ^0′^ with a perturbation amplitude of ± 10 mV.

The adsorption of the BSA protein provoked marginal modifications in the CV results in 0.3 M KCl and 0.75 mM ferrocyanide (Figure 6a). The bell-shaped pair of peaks with minimal separation indicated that the thin-layer behavior was maintained even after BSA deposition. The voltammogram displayed a slight increment in the peak separation (from 8 to 24 mV), which may be related to an increment in the ion resistance or, even, electron transfer resistance. However, once EIS was used to evaluate the state of the CNP inner surface, the changes were more evident. The response at *E*_DC_ = 0 V (outside the faradaic window) was characterized by a slight increment in the semicircle in the Nyquist plot that could suggest an increment in *R*_CNP_ (Figure 6b). By fitting both responses (before and after protein immobilization) to the simplified circuit proposed above, *R*_CNP_ showed an increase from 8.55 MΩ (relative error of fitting <1%, χ = 0.006) to 10.6 MΩ (relative error of fitting <1%, χ = 0.01). Considering that the protein radius is about 5 nm, which represents ∼10% of the tip radius, such a change is likely attributed to the partial occlusion of the tip generated by protein immobilization. Indeed, the increment in the ion transport resistance due to bulky protein adsorption has been previously reported in other nanofluidic devices.^41,42^ Additionally, the slope of the abrupt increment in −*Z*_IM_ observed in the charge saturation region exhibited a slight attenuation following BSA exposure. Within the framework of the ECM fitting, this change is reflected as a decrease in the *n*-parameter of the CPE associated with the electrical double layer, from *n* = 0.98 to *n* = 0.93. This reduction indicates a greater deviation from the ideal capacitive behavior, which may be attributed to an increase in the surface heterogeneity.^43^

**Figure 6:**
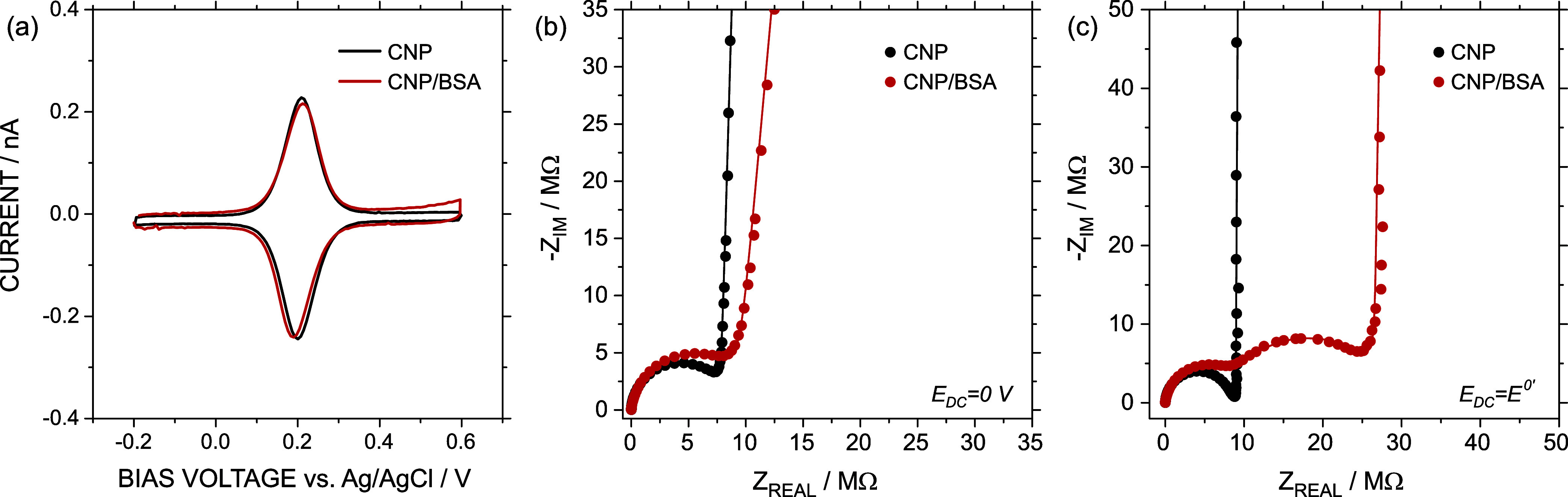
(a) CV and EIS at *E*_DC_ (b) 0 V and (c) *E* ^0′^ = 0.2 V before and after immobilization of BSA in the internal walls of the CNP. Circles and lines in figures (b, c) correspond to experimental data and ECM fitting, respectively. All the measurements were performed in 0.3 M KCl + 0.75 mM of ferrocyanide.

The trend observed in the Nyquist representation outside the faradaic window was confirmed by EIS analysis at *E*_DC_ = *E* ^0′^ (Figure 6c). In this case, the Nyquist plot not only verified the increment in *R*_CNP_ but also revealed the appearance of a second semicircle with a larger diameter. This semicircle seems to indicate an increment in the *R*_CT_, which is related to the hindrance of the redox probe reaction caused by protein adsorption on the surface. By fitting both responses to the complete electrical equivalent circuit, *R*_*CT*_ demonstrated an increase from 0.88 MΩ (relative error of fitting ∼6%, χ = 0.007) to 18.4 MΩ (relative error of fitting <2%, χ = 0.01) after BSA exposure. As a result, *R*_CT_ led to a clear separation between ion transport and electron transfer contributions in the EIS spectra; effectively, there was an increase of the characteristic time of the redox reaction. Thus, in the presence of the protein on the carbon surface, the EIS response of ferrocyanide at *E* ^0′^ resembles that obtained for the irreversible redox probe (FeCl_3_) in a pristine CNP. Further analysis by ECS and DDC can be found in Section S11, SI file.

Overall, these results suggested that small changes in the CV due to the immobilization of a nonredox active building block can be effectively amplified by EIS and ECS analysis. This finding potentially positions the mentioned techniques and developed methodologies as straightforward procedures to characterize (at least qualitatively) the CNP’s tip and the state of the surface at the nanoscale level after different functionalization steps. Notably, this characterization can be conducted without the need of transferring the system to macro-electrodes or perform ion transport experiments that require a different experimental setup. Furthermore, the established strategy could constitute a tangible path for the development of sensing protocols of nonredox bulky analytes in nanofluidic electrodes.

## Conclusions

We have presented a comprehensive analysis of the EIS response observed with CNPs, with special emphasis on the ability to decouple and quantify ion transport and redox reactions. By employing electrical equivalent circuit models alongside methods like DRT and DDC, we have demonstrated the obtention of key parameters such as CNP resistance, double-layer capacitance, redox capacitance, and the characteristic times of different processes. Transforming impedance data into the capacitance plane enables a more direct quantification of redox capacitance, which correlates with the number of moles inside the CNP. This approach offers a tangible potential toward pursuing calibration-free sensors. Moreover, we have presented that EIS can detect surface modifications even from nonredox-active species, as exemplified by the immobilization of BSA in the inner walls of the CNP, which induced noticeable changes in the Nyquist plot. This makes EIS a powerful tool for assessing functionalization strategies and optimizing device performance without requiring additional experimental setups such as the transference of the system to macroelectrodes or the filling of the nanopipette to conduct ion transport experiments. Truly, EIS and its various analytical approaches offer valuable opportunities for fundamental studies aiming to decouple ion transport from redox contributions. Beyond that, EIS has revealed strong potential for analytical applications, particularly for characterizing CNPs after treatments that alter their surface properties or tip dimensions, an area that remains challenging with conventional electrochemical techniques.

## Supplementary Material



## Notes

EIS measurements in the absence of redox probes, in macroelectrodes, at different KCl concentrations, and with a different redox probe; complementary ECS, DRT, and DDC analysis (PDF)
