# Supplementary material for: Fundamental Insights into Nanoconfined Devices by Using Electrochemical Impedance Spectroscopy
Source: Anal Chem. 2026 Jan 23;98(6):5054–65. doi: 10.1021/acs.analchem.5c07422 (PMC12921669; doi:10.1021/acs.analchem.5c07422)
Supplement: Supplementary file 1 [file ac5c07422_si_001.pdf]

Electronic Supporting Information

Fundamental Insights into Nanoconfined Devices by using Electrochemical Impedance Spectroscopy

Gregorio Laucirica[a], Danilo Echeverri[a], Gastón A. Crespo[a,b], María Cuartero[a,b,]*

[a]UCAM-SENS, Universidad Católica San Antonio de Murcia, UCAM HiTech, Avda. Andres Hernandez Ros 1, 30107, Murcia, Spain.

[b]Department of Chemistry, School of Engineering Science in Chemistry, Biochemistry and Health, KTH Royal Institute of Technology, Teknikringen 30, SE-114 28 Stockholm, Sweden.

INDEX

S1. **FESEM analysis** **3**

S2. **EIS in glassy carbon electrodes** **3**

S3. **CNP's EIS in the absence of redox probe** **4**

S4. **Cyclic voltammetry analysis at different scan rates** **5**

S5. **ECM analysis at different E_{DC}** **5**

S6. **ECS Nyquist plot for a GC electrode** **7**

S7. **Additional ECS results in CNPs** **8**

S8. **λ optimization and additional DRT and DDC results** **10**

S9. **EIS and ECS results at different redox probe concentrations** **11**

S10. **Comparison of the analytical performance of CV, EIS and ECS** **13**

S11. **ECS and DDC analysis of BSA immobilization** **14**

S12. **References** **16**

S1. FESEM analysis

The geometry of the CNPs was characterized using field-emitter scanning electron microscopy (FESEM), Zeiss Merlin VP Compact. For this purpose, a segment of the CNP (~2 cm) was cut and mounted vertically (90°) on an SEM holder. Figure S11 presents images acquired using (a) the secondary electron detector, (b) the in-lens detector, and (c) the secondary detector with the holder slightly tilted (~10°) to better capture the CNP profile. The tip radius was estimated to be approximately 60 nm, which is consistent with the value obtained from electrochemical measurements.

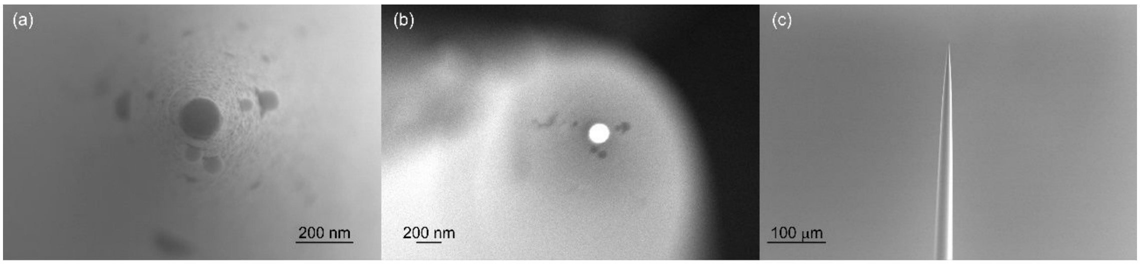

Figure S1. FESEM images of the tip aperture captured with (a) the secondary electron (magnification 86kx) and (b) in-lens (magnification 37.6kx) detectors. FESEM image of the CNP profile captured with the secondary electron (Magnification 176x).

S2. EIS in glassy carbon electrodes

In the absence of redox probe (w/o redox), Nyquist plot obtained for a glassy carbon electrode (GC) is characterized by a straight line (**Figure S1a**). In terms of electrical equivalent circuits, this trend is fitted with a resistor, accounting for the solution resistance, and a capacitor (or constant phase element), representing the electrical double layer, in series (**Figure S1b**). In the presence of equimolar concentrations of the ferro/ferricyanide, the resulting impedance response at open-circuit potential in glassy carbon electrodes demonstrates the appearance of a semicircle followed by a 45° linear trend, which is commonly modeled using a Randles circuit, which includes a resistor and capacitor in parallel, along with a Warburg element (**Figure S1b**). Here, the resistances represent the solution resistance and the charge transfer resistance at the carbon layer/electrolyte interface, the capacitance corresponds to the double-layer charging,

and the Warburg impedance accounts for the diffusion of redox species from the bulk solution to the electrode surface.[1,2]

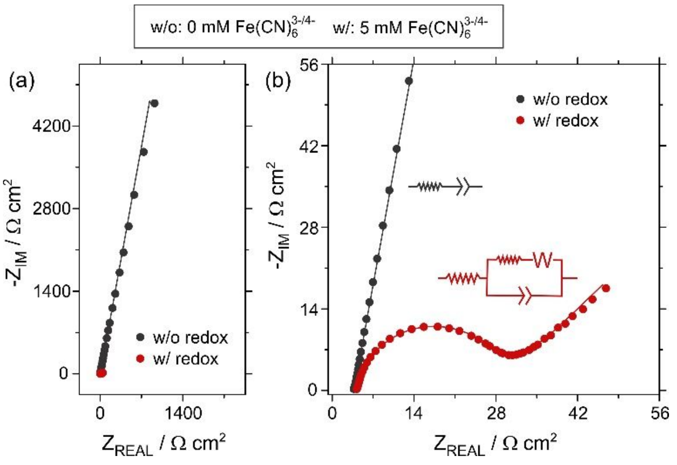

Figure S2. EIS results for a glassy carbon macroelectrode in the absence (w/o) and presence (w/) of a redox probe. Panel (a) shows the Nyquist plot in wide impedance window while, panel (b) represents a zoom-in of (a) in the region of low impedances. Points correspond to the experimental results while full lines represent their fittings to the electrical equivalent circuit models illustrated in panel (b). In all the cases, the supporting electrolyte was 0.3 M KCl. The experiment in the presence of redox probe (w/) contained equimolar concentration (5 mM) of ferrocyanide and ferricyanide. Both measurements were performed at the open circuit potential, 10 mV of amplitude, and from 1 Hz to 100 kHz. The counter electrode and reference electrode were a platinum rod and Ag/AgCl wire, respectively. Impedance signals were multiplied by the geometrical glassy carbon area (0.071 cm^2).

S3. CNP's EIS in the absence of redox probe

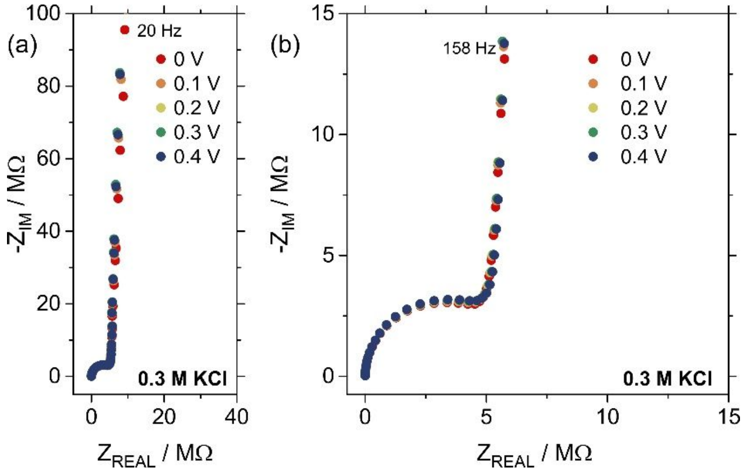

Figure S3. EIS results for a CNP in the absence of any redox probe.at different E_{DC}. Panel (a) shows the Nyquist plot in wide frequency window (from 10^6 to 20 Hz) while, panel (b) represents a zoom-in of (a) in the region of moderate/high frequencies (from 10^6 to 158 Hz). In all the cases, the supporting electrolyte was 0.3 M KCl.

S4. Cyclic voltammetry analysis at different scan rates

The electrochemical response of open CNPs is sensitively affected by the properties of the redox probe inside the nanofluidic device and the ion transport through the nanotip. As demonstrated in our previous work, the confined environment of the nanopipette leads to a thin-layer behavior of the redox probe in the solution placed inside the CNP.[3] This means that in the typical timescale for electroanalytical experiments (from very few seconds to a few minutes), the redox reaction of the probe can evolve exhaustively. This involves the total consumption of the analyte, which is evidenced in the electrochemical response. For example, a CV conducted in 0.3 M KCl as supporting electrolyte containing 0.10 mM ferrocyanide as the redox probe showed the typical bell-shaped peaks characteristic of thin-layer systems (**Figure S4a**). In addition, the peak potential separation (ΔE_p) being close to 0 mV, the linear relationship of the peak current with the scan rate (r^2=0.998), and the independence of the voltammetric charge Q (black points) with the scan rate (less than 5% of variation in the evaluated range) confirm the thin-layer behavior (**Figure S4b**). It is worth mentioning that Q in **Figure S4b** corresponds to the anodic charge, although the variation compared to the cathodic one was <1 % for all the scan rates, as expected for reversible redox couples.

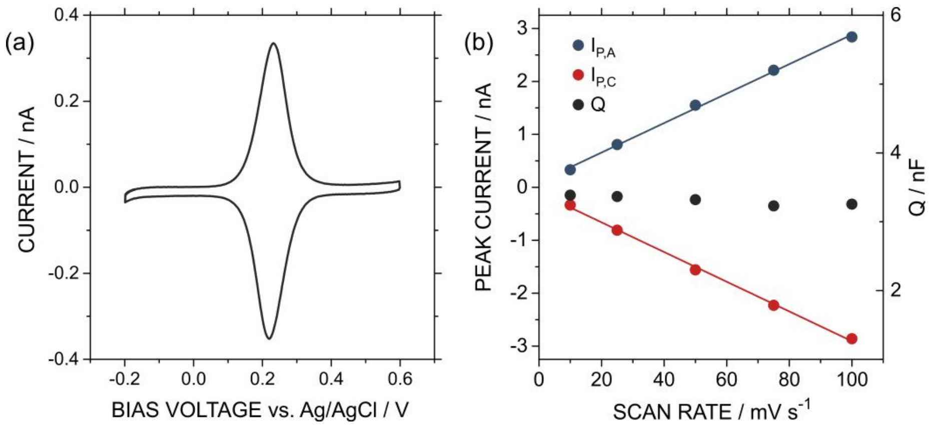

Figure S4. (a) CV obtained in 0.10 mM ferrocyanide solution in 0.3 M KCl at 10 mV s^{-1}. (b) Peak current and charge magnitudes as a function of scan rate. $I_{P,A}$, $I_{P,C}$ and Q correspond to the anodic peak current, cathodic peak current, and voltammetric charge, respectively.

S5. ECM analysis at different E_{DC}

The trends observed for C_{REDOX} and R_{CT} were highly sensitive to the applied bias voltage. This is because the E_{DC} magnitude determines the redox probe concentrations, and the faradaic contribution to EIS measurements sensitively depends on the ratio between the oxidized and reduced species. This aspect can be further illustrated by analyzing the expressions for each magnitude defined for the case of confined electro-domains (a complete derivation of these equations can be found in the available literature)[4,5]:

$$R_{CT} = \frac{RT}{F^2 V k_s} * \frac{1}{(\alpha c_O \varphi^{-\alpha} + (1-\alpha) c_R \varphi^{1-\alpha})} \quad (1)$$

$$C_{REDOX} = \frac{F^2 V}{RT} * \frac{(\alpha c_O \varphi^{-\alpha} + (1-\alpha) c_R \varphi^{1-\alpha})}{(\varphi^{-\alpha} + \varphi^{1-\alpha})} \quad (2)$$

with

$$\varphi^{-\alpha} = exp^{\frac{-\alpha F(E-E^{0\prime})}{RT}} \quad (3)$$

$$\varphi^{1-\alpha} = exp^{\frac{(1-\alpha)F(E-E^{0\prime})}{RT}} \quad (4)$$

where F, c_R, c_O, V, R, k_s, α and T are the Faraday constant, concentration of the oxidized probe, concentration of the reduced probe, volume, ideal gas constant, the rate constant at the standard potential (units of s^{-1}), charge transfer coefficient, and temperature, respectively. Equations (1) and (2) explain the trend observed in **Figures 4e** and **4f** for R_{CT} and C_{REDOX} in terms of E_{DC}. Specifically, when $E_{DC}=E^{0'}$ ($\varphi^{-\alpha}$ and $\varphi^{1-\alpha}=1$), both redox states of the probe were present at appreciable concentrations (indeed $c_O=c_R$), the reaction rate was maximized and, consequently, C_{REDOX} and R_{CT} displayed their maximum and minimum values.[2] Therefore, while it is possible to obtain information on the ion transport by measuring outside the faradaic window (e.g., E_{DC}=0 V), critical information regarding the redox performance is provided by the measurements at $E_{DC}=E^{0'}$. For this reason, EIS measurements were always evaluated inside and outside the faradaic window.

Table S1. ECM fitting results at different E_{DC}.

	E_{DC} / V	R_{CNP} / MΩ	R_{CT} / MΩ	C_{CNP} / nF	$Y_{0,DL}$ / nF	n_{DL}	$Y_{0,REDOX}$ / nF	n_{REDOX}
OUT	-0.1	8.59	-	0.011	0.87	0.96	-	
	0	8.61	-	0.011	0.81	0.97	-	
	0.05	8.60	109	0.011	0.86	0.96	-	
INSIDE	0.1	8.64	13.2	0.011	0.94	0.99	0.41	0.99
	0.125	8.66	4.18	0.011	0.95	1	1.35	0.97
	0.15	8.62	1.37	0.010	0.95	1	3.71	0.98
	0.17	8.58	0.72	0.010	0.95	1	7.54	0.99
	0.19	8.55	0.47	0.010	0.95	1	13.40	0.99
	0.2	8.49	0.48	0.010	0.95	1	16.80	0.99
	0.21	8.51	0.40	0.010	0.95	1	19.70	0.99
	0.22	8.48	0.40	0.010	0.95	1	22.00	0.99
	0.23	8.44	0.42	0.010	0.95	1	21.60	0.99
	0.25	8.54	0.41	0.010	0.95	1	17.80	0.99
	0.275	8.60	0.67	0.011	0.95	1	9.88	0.99
	0.3	8.64	1.30	0.011	0.95	1	4.32	0.98
	0.35	8.63	11.5	0.011	0.93	0.98	0.58	0.98

	0.4	8.57	44.6	0.010	0.78	0.97	0.14	0.89
OUT	0.5	8.57	-	0.010	0.83	0.97	-	-

Table S2. Ion resistance obtained at different KCl concentrations.

KCl concentration / M	R_{CNP} / MW
0.3	4.05
0.55	2.38
0.75	1.79
1	1.44
1.5	1.02

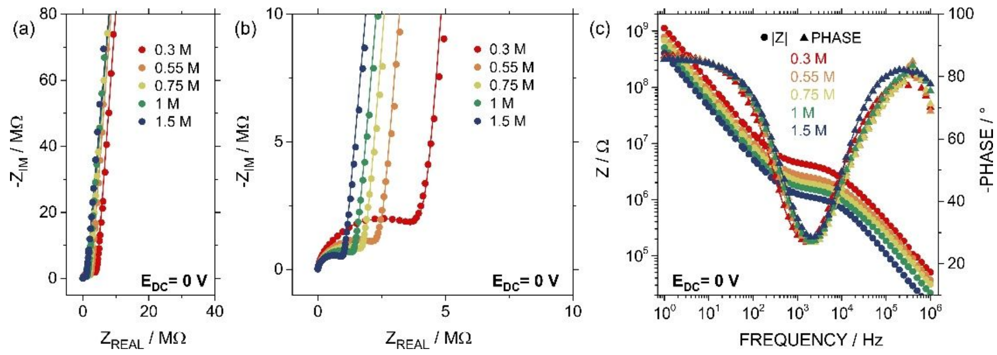

Figure S5. (a) Nyquist (and zoomed-in), and (b) Bode plots for a CNP in 0.77 mM ferrocyanide and different KCl concentrations. EIS measurements were performed at E_{DC}= 0 V (outside faradaic window). All the experimental results (circles) are overlapped with the ECM fitting (lines).

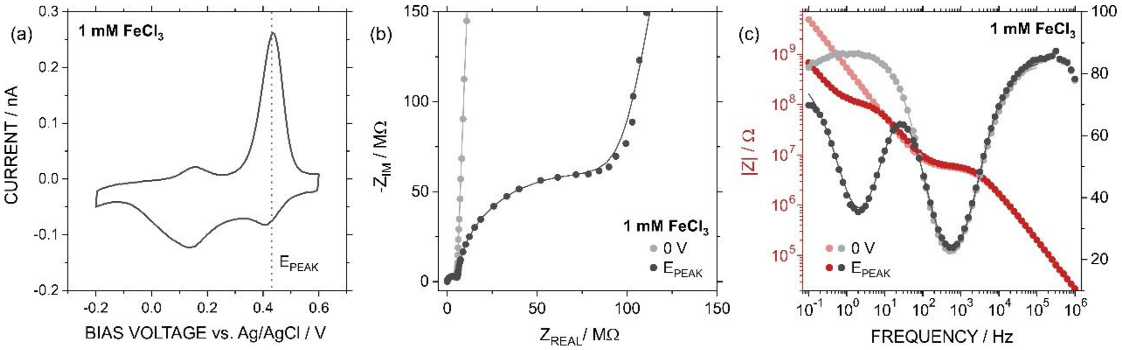

Figure S6. (a) CV, (b) Nyquist, and (c) Bode plots for a CNP in 0.3 M KCl (red) + 1 mM $FeCl_3$ solution. EIS measurements were performed at E_{DC} 0 V and 0.42 V (E_{PEAK}). In Figures (b) and (c), all the experimental results (circles) are overlapped with the ECM fitting (lines). For the present case, ECM analysis evidences an R_{CT}□111 MW, which is approximately 20 times higher than $R_{CNP.}$

S6. ECS Nyquist plot for a GC electrode

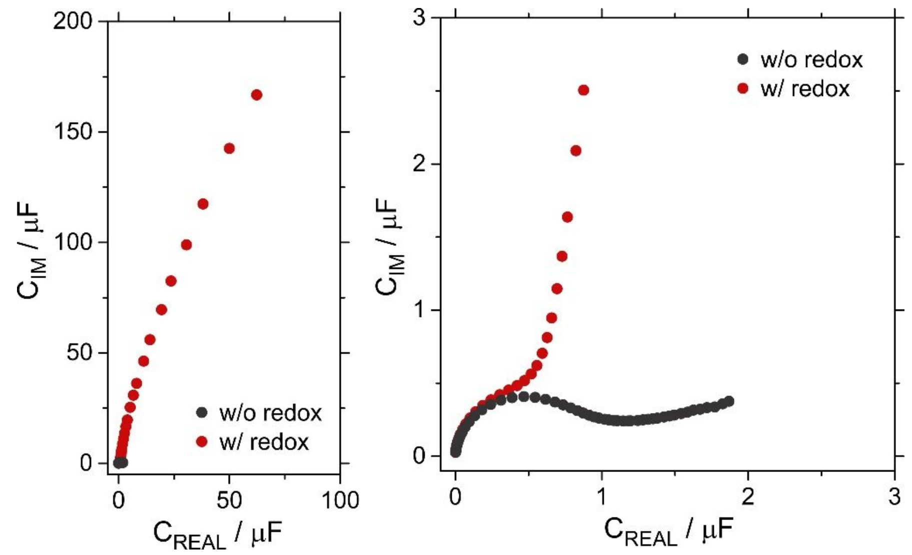

Figure S7. (a) Nyquist (and zoomed-in) for a glassy carbon working electrode (0.071 cm^2) in 0.3 M KCl (black circles) and 0.3 M KCl + equimolar 5 mM ferrocyanide: 5 mM ferricyanide (red circles) solutions. EIS measurements were performed at the open circuit potential, 10 mV of amplitude, and from 1 Hz to 100 kHz. The counter electrode and reference electrode were a platinum rod and Ag/AgCl wire, respectively. EISs are available in **Figure S1**.

S7. Additional ECS results in CNPs

Beyond the C_{IM} vs. C_{REAL} (analogue to the Nyquist plot, **Figure S7a**), ECS results can be analyzed in terms of C_{REAL} or C_{IM} vs. frequency plots (analogue to the Bode Plot). The C_{REAL} vs. f graph was characterized by an asymptotic trend for C_{REAL} ($f\rightarrow 0$) that increased in magnitude as E_{DC} approached $E^{0'}$ (**Figure S7b**). The analysis of C_{REAL} ($f\rightarrow 0$) vs. E_{DC} exhibited a maximum value when $E_{DC} \sim E^{0'}$. As was explained in the main text, the subtraction of the ionic component to such maximum value enables the decoupling of C_{REDOX} from C_{DL}.

C_{IM} vs. f plot provides information of the characteristic times τ ($\tau = 1/2\pi f_{max}$) of the capacitive phenomena involved in the system (**Figure S7c**). These graphs exhibited a maximum that indicated the capacitor's characteristic frequency (f_{max}), but with the magnitude and position being highly sensitive to E_{DC}. When the E_{DC} was outside the faradaic window, the position and magnitude of the maximum remained constant with E_{DC}. Consequently, it enabled the determination of the characteristic time τ ($\tau = 1/2\pi f_{max}$) of the electrical double-layer charging (τ_{IONIC}). On the other hand, when E_{DC} approaches $E^{0'}$, the maximum position was shifted towards lower f values, indicating an increase of τ for reaching the charge saturation condition. At $E_{DC} \sim E^{0'}$, f_{max} acquired the lowest value corresponding to the characteristic frequency of the absolute consumption of the analyte in the thin-layer domain. Considering this, the τ vs. E_{DC} plot exhibited a maximum for $E_{DC} \sim E^{0'}$ with values of 6 ms and 0.2 s for τ_{IONIC} and the characteristic time of the thin-layer redox reaction (τ_{REDOX}), respectively (**Figure S8**). Notably, the trend of τ_{REDOX} was very similar to that observed for C_{REAL} ($f\rightarrow 0$).

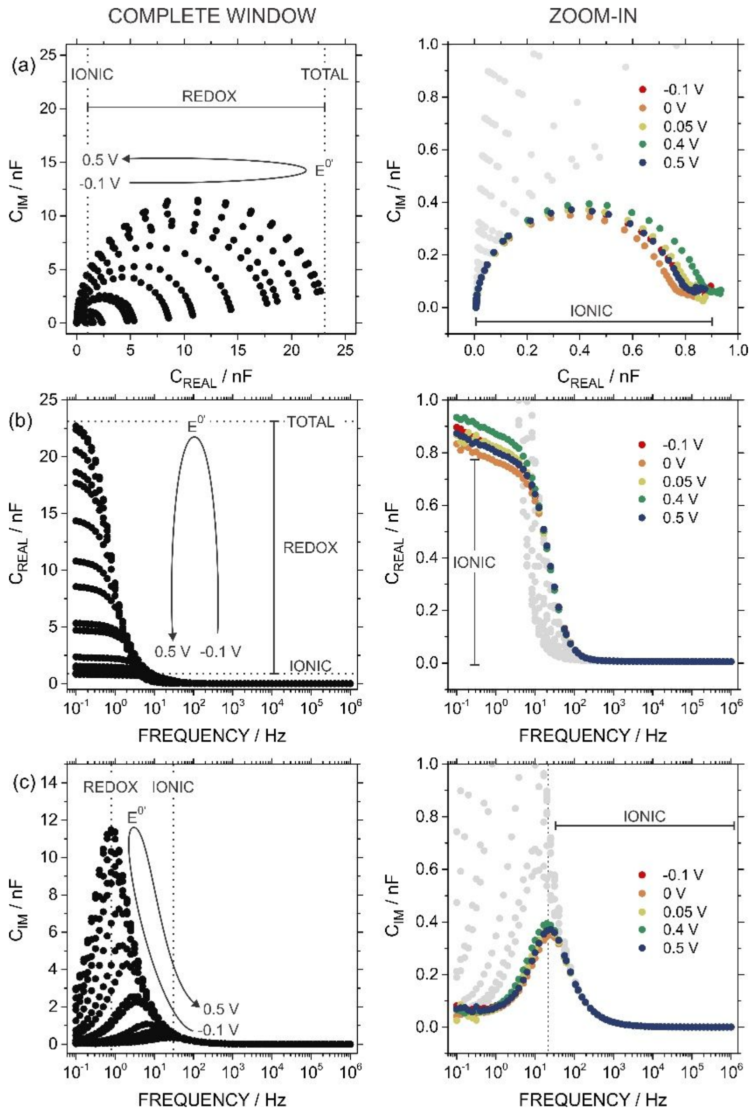

Figure S8. (a) ECS Nyquist plot at different E_{DC} ranging from -0.1 V to 0.5 V. (b) C_{REAL} and (c) C_{IM} in terms of the frequency for different E_{DC} values. In all the cases, the right panels were added to visualize ECS response under only ionic contribution. All the measurements were conducted in 0.3 M KCl and 0.10 mM ferrocyanide.

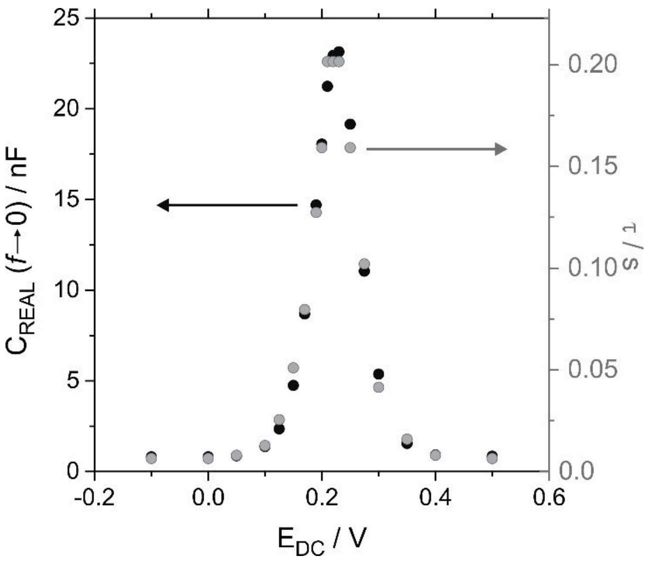

Figure S9. C_{REAL} (f→0) and τ in terms of E_{DC}. All the measurements were conducted in 0.3 M KCl and 0.10 mM ferrocyanide. In all the cases, the plots obtained at 0 V and 0.5 V overlap (further details are available in the ESI).

S8. λ optimization and additional DRT and DDC results

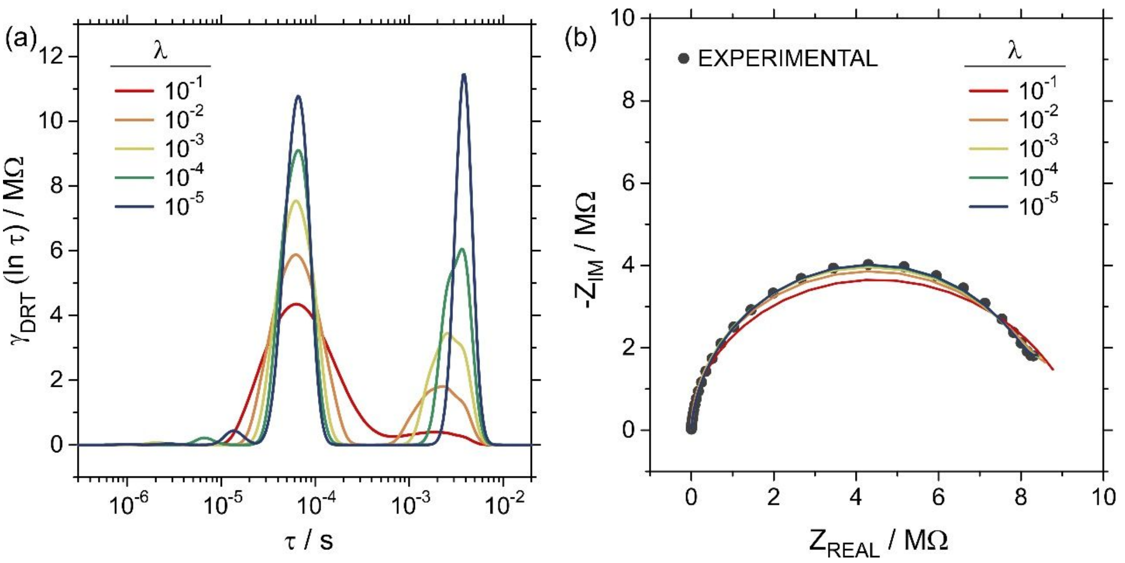

Figure S10. (a) Comparison of DRT results obtained with different regularization parameter λ. (b) Experimental (circles) and predicted Nyquist plots by DRT analysis at different λ. EIS measurement was performed at E_{DC}=0 V in a solution composed of 0.3 M KCl and 0.1 mM ferrocyanide.

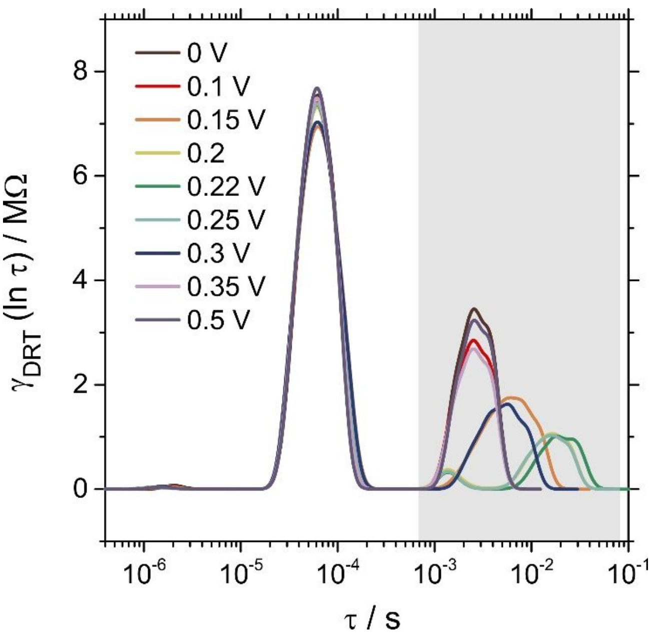

Figure S11. (a) DRT spectra in terms of τ. All the experiments were performed in 0.3 M KCl and 0.1 mM ferrocyanide.

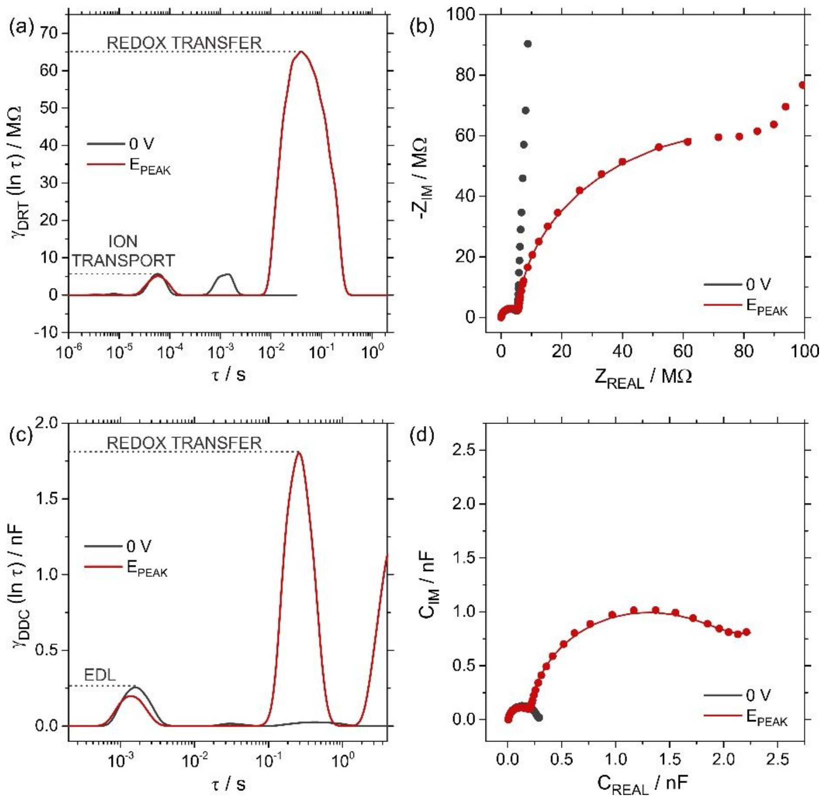

Figure S12. EIS results: (a) DRT and (b) Nyquist plots. ECS results: (c) DDC and (d) ECS Nyquist plots. EIS measurements were performed at E_{DC} 0 V and 0.42 V (E_{PEAK}). for a CNP in 0.3 M KCl (red) + 1 mM $FeCl_3$ solution. DRT and DDC results were obtained with λ=0.001.

S9. EIS and ECS results at different redox probe concentrations

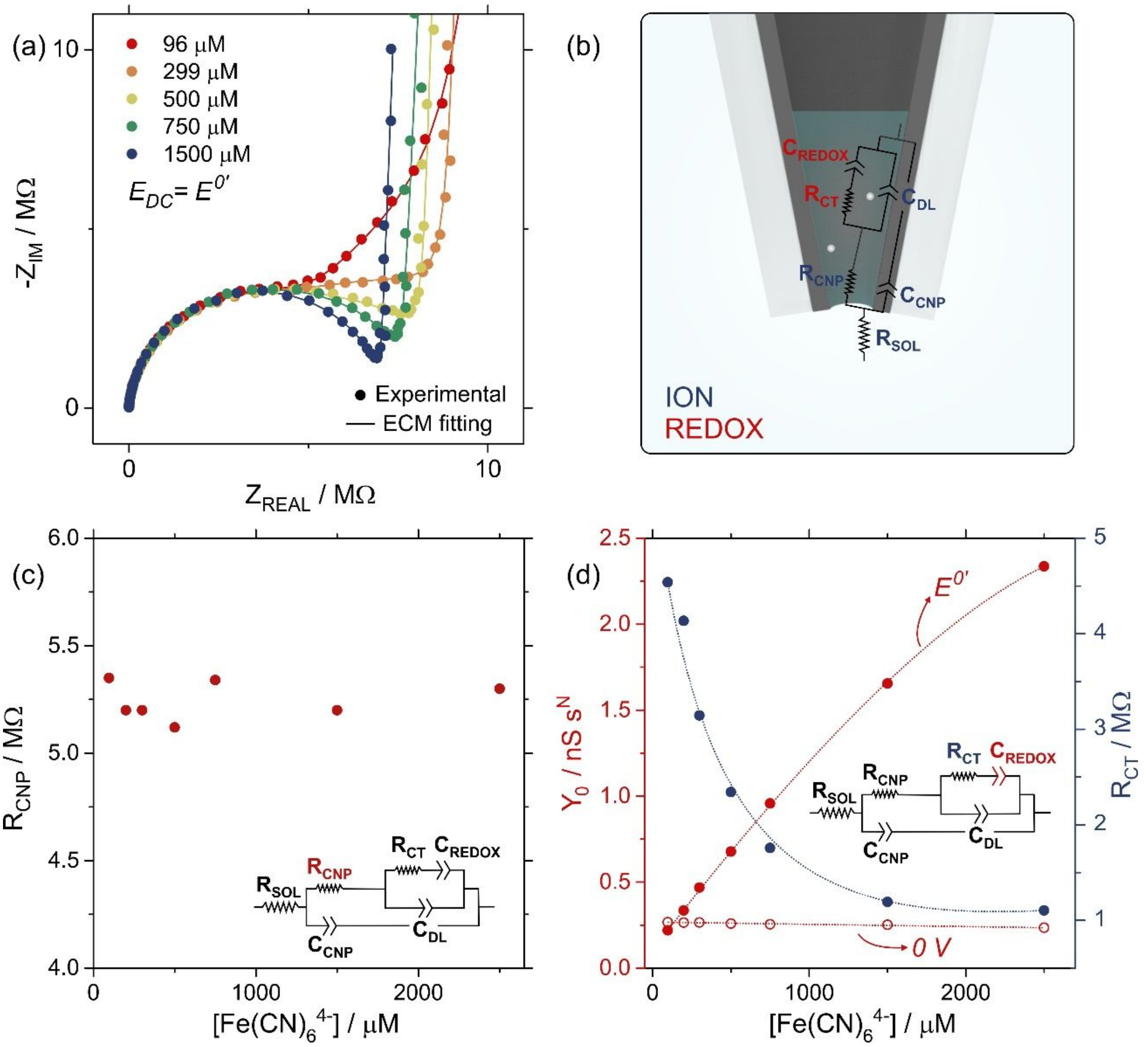

Figure S13. (a) Nyquist plot at different ferrocyanide concentrations. Circles and full lines represent the experimental results and the ECM fitting, respectively. (b) Scheme of the ECM employed for the EIS fitting. (c) R_{CNP} obtained from the ECM fitting at the different ferrocyanide concentrations. (d) Redox capacitance (in terms of Y_0) and R_{CT} obtained from the ECM fitting at the different ferrocyanide concentrations. Considering that N factor of CPE was between 0.96-1, Y_0□C_{REDOX}.

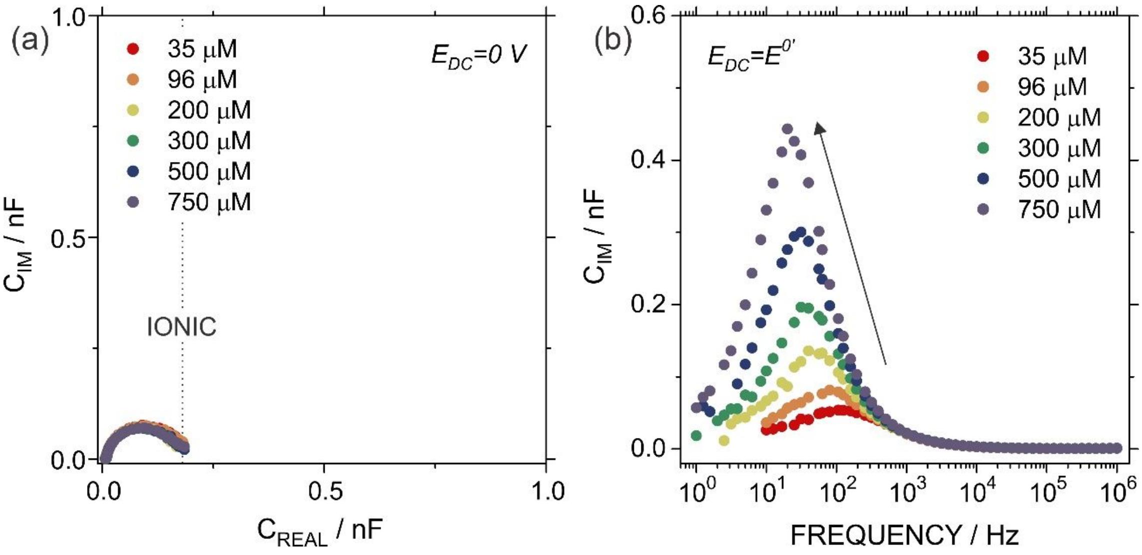

Figure S14. (a) ECS Nyquist plots at E_{DC}= 0 V (outside faradaic window) for increasing ferrocyanide concentrations. (b) C_{IM} in terms of the frequency at E_{DC}= $E^{0'}$ for increasing ferrocyanide concentrations. All the measurements were conducted in 0.3 M KCl as the supporting electrolyte.

S10. Comparison of the analytical performance of CV, EIS and ECS

Figures S15a-c compare CV, EIS, and ECS records at increasing concentrations of the redox probe aiming at a comparison of the features provided by each one. Compared to the results shown in **Figure 5** of the main manuscript, the sensitivity of the device was approximately 7 times higher. This is because the volume inside the CNP was higher and, therefore, the increase in the total number of redox probe moles.[3]

The peak current, |*Z*| at 40 Hz (in the charge saturation region) and C_{REDOX} were selected as the analytical parameters for CV, EIS, and ECS, respectively (**Figures S15d-f**). Notably, calculating C_{REDOX} from the ECM fitting of EIS represented a challenging process: small variations in the circuit parameters led to significant changes in this value, particularly at very low concentrations of the redox probe. For example, for a concentration of 5 µM, variations of 2.5% in C_{DL} resulted in changes up to 50% and 35% in the magnitudes of R_{CT} and C_{REDOX}, respectively. For a concentration of 50 µM, variations of 2.5% in C_{DL} only produced changes of 2-3% in the magnitudes of R_{CT} and C_{REDOX}. For this reason, only the estimation of C_{REDOX} by ECS was included in the analysis. Overall, the compared methods in **Figures S15d-f** presented similar capabilities regarding the minimal discernible concentration of the redox probe. Also, the transformation of EIS into ECS made it possible to determine the volume inside the CNP. For this case, the volumes estimated from the ECS and CV analysis slopes were 7.1 ± 0.4 pL and 6.3 ± 0.3 pL, respectively.

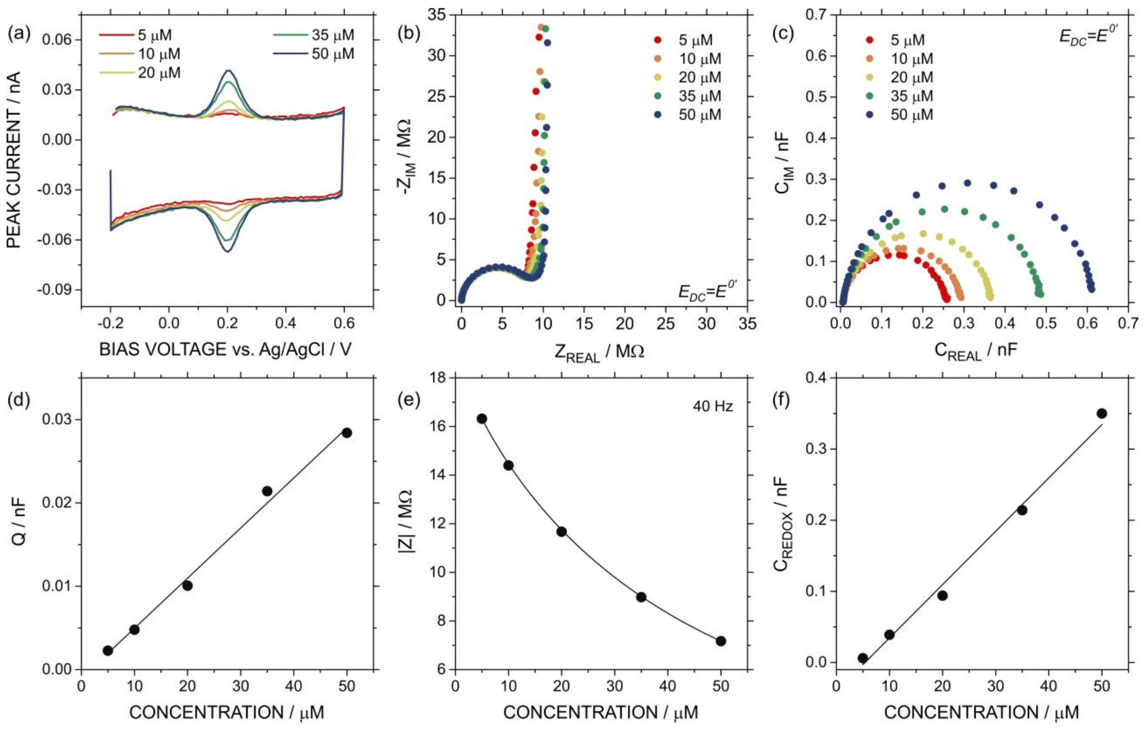

Figure S15. (a) CV, (b) EIS (E_{DC}=$E^{0'}$), and (c) ECS (E_{DC}=$E^{0'}$) at increasing ferrocyanide concentrations. Analysis of (c) Q (extracted from the CVs), (d) |Z| at 40 Hz (extracted from EIS), and (e) C_{REDOX} (extracted from ECS) in terms of the redox probe concentration. All the measurements were conducted employing 0.3 M KCl as the supporting electrolyte.

S11. ECS and DDC analysis of BSA immobilization

The effect of the BSA immobilization was further assessed using ECS (**Figure S16a**). The ECS Nyquist plot at E_{DC}=$E^{0'}$ exhibited a reduction in the semicircle diameter (total capacitance), indicating a drop in the accumulated charge. This behavior can be ascribed to two factors. First, tiny variations in the CNP inner volume may occur due to practical aspects (e.g., solution exchanges during measurement, modification, and cleaning of the CNP). However, the minimal change in total capacitance at 0 V (outside the faradaic window, see zoomed region in **Figure S16b**) suggests that the volume change alone does not fully explain the observed variation. Another plausible explanation involves an accentuation of the effect of the surface electrical potential at the CNP tip because of a reduction in the effective size (in agreement with the increment in R_{CNP}), together with an increase in surface charge caused by the adsorption of negatively charged BSA. The decrease in total charge from redox reactions is likely originated from a stronger surface electrical potential, which repels charged redox couples, as previously suggested for other systems.[6,7]

In addition to the variation of the capacitance values, **Figure S16b** demonstrates that, like the EIS results, the deconvolution of ion- and redox-related capacitances could be observed after protein immobilization in measurements conducted within the faradaic window (full red circles in **Figure 16b**). To further clarify this point, ECS data were transformed into DDC plots (**Figure 16c**). In measurements at 0 V (outside the faradaic window), DDC deconvolution revealed a peak at approximately 0.002 s before and after CNP modification, corresponding to the double-layer charging (**Figure 16d**). Conversely, for the measurements at E_{DC}=$E^{0'}$, while only a single peak at 0.08 s was present before

BSA adsorption, this was found to led to two distinct peaks at 0.002 s and 0.2 s. Our findings indicated that the surface modification shifts the redox reaction's characteristic time from 0.08 s to 0.2 s, promoting a clear separation of ionic and redox process timescales, as evidenced in both ECS and DDC results.

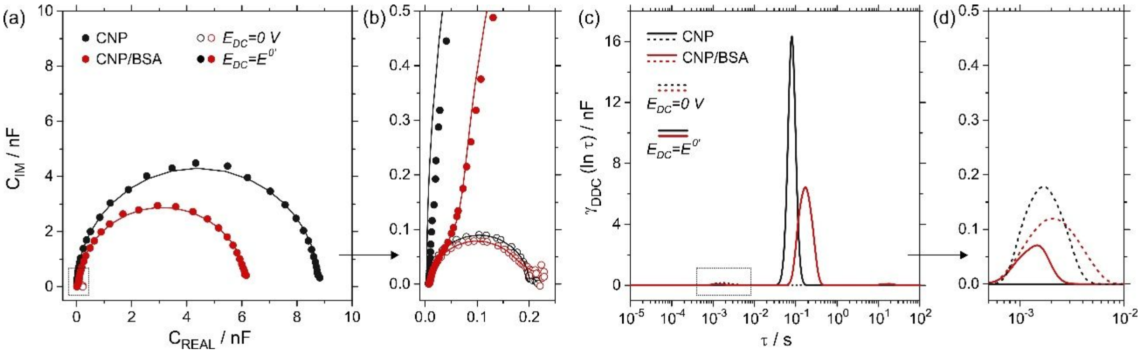

Figure S16. (a)-(b) ECS and (f)-(g) DDC at E_{DC} 0 V and $E^{0'}$=0.2 V before and after immobilization of BSA. Panel (c) and (d) correspond to the zoom-in of the region marked with a dashed-line box in figures (a) and (c), respectively. In Figure (a)-(b), empty circles and lines correspond to experimental data and ECS fitting from DDC results, respectively. In Figure (c)-(d), a regularization parameter of 0.001 was employed. All the measurements were performed in 0.3 M KCl + 0.75 mM of ferrocyanide.

S12. References

(1) Lazanas, A. C.; Prodromidis, M. I. Electrochemical Impedance Spectroscopy─A Tutorial. *ACS Meas. Sci. Au* **2023**, *3* (3), 162–193. https://doi.org/10.1021/acsmeasuresciau.2c00070.

(2) Bard, A. J.; Faulkner, L. R. *Electrochemical Methods. Fundamentals and Applications*, 2nd ed.; Harris, D., Swain, E., Eds.; Wiley: USA, 2001.

(3) Laucirica, G.; Crespo, G. A.; Cuartero, M. Thin-Layer Behavior in Carbon Nanopipettes. Understanding the Iontronic-Electronic Contributions. *Anal. Chem.* **2025**. https://doi.org/10.1021/acs.analchem.5c02834.

(4) Laviron, E. A.C. Polarography and Faradaic Impedance of Strongly Adsorbed Electroactive Species. *J. Electroanal. Chem. Interfacial Electrochem.* **1979**, *97* (2), 135–149. https://doi.org/10.1016/S0022-0728(79)80057-1.

(5) Prieto, F.; Rueda, M.; Hidalgo, J.; Martínez, E.; Navarro, I. Electrochemical Impedance Spectroscopy Study of a Surface Confined Redox Reaction: The Reduction of Azobenzene on Mercury in the Absence of Diffusion. *Electrochim. Acta* **2011**, *56* (23), 7916–7922. https://doi.org/10.1016/j.electacta.2010.12.061.

(6) Bae, J. H.; Wang, D.; Hu, K.; Mirkin, M. V. Surface-Charge Effects on Voltammetry in Carbon Nanocavities. *Anal. Chem.* **2019**, *91* (9), 5530–5536. https://doi.org/10.1021/acs.analchem.9b00426.

(7) Liao, T.; Li, X.; Tong, Q.; Zou, K.; Zhang, H.; Tang, L.; Sun, Z.; Zhang, G.-J. Ultrasensitive Detection of MicroRNAs with Morpholino-Functionalized Nanochannel Biosensor. *Anal. Chem.* **2017**, *89* (10), 5511–5518. https://doi.org/10.1021/acs.analchem.7b00487.
